# Supplementary material for: Whole-genome resequencing of wild and domestic sheep identifies genes associated with morphological and agronomic traits
Source: Nat Commun. 2020 Jun 4;11:2815. doi: 10.1038/s41467-020-16485-1 (PMC7272655; doi:10.1038/s41467-020-16485-1)
Supplement: Supplementary file 1 — Supplementary information [file 41467_2020_16485_MOESM1_ESM.pdf]

Whole-genome resequencing of wild and domestic sheep identifies genes associated with morphological and agronomic traits

Li *et al.*

Supplementary Methods

1. Validation of the called SNPs

We used two approaches to examine the accuracy of the called SNPs. For the dbSNP-based test, we estimated the number of concordant sites with the same genomic positions between the called SNPs and the *Ovis aries* dbSNP v.151 (NCBI) for each of the 248 individuals. This number was then divided by the total number of called SNPs in the corresponding individual to obtain its validation rate. For the chip-based test, we first extracted the common SNP loci shared between the called SNPs and those on the Ovine Infinium HD BeadChip array (~600K SNPs) (Illumina, San Diego, CA) for 223 samples with available chip data. We then compared the genotypes of the common loci in the called SNPs and Ovine Infinium HD chips for each individual. The number of loci with the same genotypes was divided by the number of common loci in the corresponding individual to give its accuracy rate. The chip-based SNPs were filtered using the same criteria as those adopted in this study, and the genomic locations of the SNPs were adjusted according to the sheep reference genome assembly Oar v.4.0.

2. Definition of genomic SVs and CNVs

We detected copy number variations (CNVs) with sizes between 176 bp and 224.6 kb and structural variations (SVs, including insertions (INS), deletions (DEL), duplications (DUP) and translocations (TRA)) with sizes between 50 bp and 984.0 kb. Two adjacent CNVs were identified as the same one if their start and end positions

varied less than 1 kb, and the overlapping regions were more than 50% of the total size[1]. The numbers of shared and unique CNVs and SVs for landrace, improved sheep and Asiatic mouflon were shown in Venn diagram.

3. Haplotype-based selection scans

We estimated the integrated Haplotype Score (*iHS*) across the genomes of Asiatic mouflon and different groups of domestic sheep populations using the Selscan v1.2.0 (ref. [2]). First, we retrieved the information on ancestral alleles from the file sheep.oarv3.1.ancestral_alleles[3] and eliminated the sites without information on their status of the ancestral alleles. We then phased SNPs and inferred haplotype for each chromosome in each group using the SHAPEIT v2.r904 (ref. [4]). We subsequently performed *iHS* scans for each group based on the haplotype data, with 50-kb sliding windows and 25-kb stepwise, a recombination rate of 1 cM Mb^-1 (ref. [5]) and Selscan default parameters --max-extend 1000000 --max-gap 200000 --cutoff 0.05. The outputs of unstandardized *iHS* scores for individuals SNPs were normalized over chromosome using the script norm implemented in Selscan with parameter --bins 100. Finally, we computed the proportion of SNPs with normalized |*iHS*| > 2 in non-overlapping windows and identified the signals of positive selection as those windows above the 95th percentile of genome-wide distribution in the tested group.

4. HKA tests

To identify the selected regions associated with domestication, we employed the

Hudson-Kreitman-Aguadé (HKA) test[6,7] to compare the polymorphism and divergence within a fixed size (i.e., 50-kb window) of genomic region relative to a neutral expectation based on the variations in the whole genome. Let S_i^A denote the observed number of polymorphic sites in the window *i* from the five old landraces of domestic sheep, S_i^B the observed number of polymorphic sites in the window *i* from the outgroup Asiatic mouflon, and D_i the observed number of differences in the window *i* between old landraces and Asiatic mouflon. Moreover, let $E(S_i^A)$ denote the expected number of polymorphic sites in the window *i* from old landraces, $E(S_i^B)$ the expected number of polymorphic sites in the window *i* from Asiatic mouflon, and $E(D_i)$ the expected number of differences in the window *i* between old landraces and Asiatic mouflon. Then, the chi-squared test statistic could be measured as follows:

$$\chi^2 = [S_i^A - E(S_i^A)]^2/E(S_i^A) + [S_i^B - E(S_i^B)]^2/E(S_i^B) + [D_i - E(D_i)]^2/E(D_i)$$

To detect the selective signals associated with particular traits, we used the HKA test[6,8] to search for genomic regions that have low levels of divergence among domestic sheep populations but normal levels of divergence between domestic sheep and the outgroup (i.e., Asiatic mouflon). Let D_i^A and $E(D_i^A)$ denote the observed and expected numbers of differences in a fixed size (i.e., 50-kb window) of genomic region among the domestic sheep populations tested for a particular trait, and D_i^B and $E(D_i^B)$ the observed and expected number of differences in the same 50-kb window between domestic sheep and Asiatic mouflon. Then, the chi-squared test statistic could be calculated using the following equation:

$$\chi^2 = [D_i^A - E(D_i^A)]^2/E(D_i^A) + [D_i^B - E(D_i^B)]^2/E(D_i^B)$$

For all the HKA tests, we calculated the chi-squared statistic in 50-kb sliding windows and shift of 25-kb across genomes to find potential selective sweeps deviated from genome-wide neutral expectation. Two loci were analyzed each time, one was the 50-kb window taken from the tested genome and the other was the virtual neutral 50-kb window in terms of the average value of nucleotide statistics in the whole genome. After application of the HKA test for each sliding window, the χ^2 statistic used for measuring the goodness-of-fit was obtained and subsequently considered to infer the putative selective sweeps.

5. Experimental validation of SNPs and CNVs

We verified 68 SNPs located within 33 important putatively selected genes (e.g., *ARHGEF4*, *HOXD1*, *KRT72, BMPR1B, RXFP2, PDGFD* and *BCO2*) by PCR amplifications and Sanger sequencing of 5–22 individuals from 21 breeds (Supplementary Table 1). The primers used for the PCRs were designed with the Primer Premier 5 (ref. [9]; Supplementary Table 1). The PCR reactions were performed in a total volume of 30 μl (i.e., 15 μl of 2× Super PCR Mix (Beijing Genomics Institute, Beijing, China), 1 μl (10 pmol μL^{-1}) of each primer, 1 μl of template DNA (30 ng μL^{-1}), and 12 μl ddH$_2$O) under the thermocycling condition of an initial step at 95 °C for 15 min, followed by 35 cycles at 95 °C for 30 sec, 60 °C for 90 sec, and 72 °C for 30 sec, and then a final extension at 72 °C for 7 min. Following the PCRs,

the amplification products were sequenced on the ABI 3730XL DNA Analyzer (Life Technologies, Carlsbad, CA, USA), and the sequencing peaks were manually checked with the BioEdit[10]. Subsequently, genotypes obtained from the Sanger sequencing were compared with those inferred by the GATK and Samtools pipelines from the whole-genome resequencing data for the same individuals.

Moreover, we validated 14 randomly selected CNVs (e.g., eight deletions and six duplications; Supplementary Data 8) by quantitative real-time PCR (qPCR). Firstly, we extracted DNA from tested and control samples (Supplementary Data 8) for whole-genome resequencing by using the DNeasy 96 Blood & Tissue Kit (Qiagen, Dusseldorf, Germany) and designed primers surrounding the deletions and within the duplications (Supplementary Data 8) using the Primer Premier 5 (ref. [9]). We then genotyped deletions by regular PCRs and examined the lengths of the PCR products by gel assays. For heterozygous deletions (i.e., deletions exhibiting different product lengths in the gel assays) and all duplications, we measured their relative copy numbers using qPCR. The qPCR was performed on the QuantStudio™ 6 Flex Real-Time PCR System (Life Technologies, Carlsbad, CA, USA) using SYBR Green (Promega, Madison, WI, USA) and the *DGAT2* gene was used as an internal reference gene. The reaction was in a total of 10 μl volume consisting of 5 μl of 2× GoTaq® qPCR Master Mix (Promega, Madison, WI, USA), 0.1 μl of CXR reference dye (Promega, Madison, WI, USA), 0.3 μl (10 pmol μL^{-1}) of each primer, 2 μl of template DNA (20 ng μl^{-1}) and 2.3 μl of water. The thermocycling condition included an initial

step at 95 °C for 10 min, followed by 40 cycles of 95 °C for 15 s and 60 °C for 1 min, and then a final extension at 72 °C for 10min.

After the qPCR, we used the $\Delta\Delta C_T$ method[11] to estimate the copy numbers. We calculated the $\Delta\Delta C_T$ value based on the equation: $\Delta\Delta C_T = (C_{T\_target} - C_{T\_DGAT2})_{sample\_A} - (C_{T\_control} - C_{T\_DGAT2})_{sample\_B}$, in which C_T is the cycle threshold, sample A the tested individual and sample B the control individual[11]. We also measured the standard deviation of the $\Delta\Delta C_T$ value, which is the same as the standard deviation of the ΔC_T value and calculated using the formula: $s = (s_1^2 + s_2^2)^{1/2}$, where s_1 is the variance of the target C_T value and s_2 the variance of the reference C_T value[11]. Finally, the concordance of calling results obtained from the CNVnator and DELLY and the relative copy number from the qPCR were evaluated based on the $\Delta\Delta C_T$ results. The $\Delta\Delta C_T$ results between 1.414 and 2.449 were considered to most likely represent a normal copy number of 2 (ref. [12]).

Supplementary Notes

1. SNP calling, mapping rate and experimental validation for SNPs and CNVs

For the 20 domestic sheep from the Middle East and Africa with one individual per breed (Supplementary Data 1), we obtained a total of 32,170,706 and 42,033,337 SNPs with SAMtools and GATK, respectively, of which 26,977,390 (17,674,936 with filtering) were identified by both methods (Supplementary Data 3). Only the SNPs exist in the 228 sample set were retained for the population structure, demographic

history (e.g., pairwise sequential Markovian coalescent (PSMC)), and domestication and improvement (e.g., global F_{ST}) associated selection analyses.

We obtained higher mapping rates for Asiatic mouflon and domestic sheep (99.04%) than those found in other wild sheep species (e.g., European mouflon *O. musimon*, 91.24%; argali *O. ammon*, 91.30%; and bighorn sheep *O. canadensis*, 95%)[13,14], suggesting a high level of genomic similarity and close phylogenetic relationship between Asiatic mouflon and domestic sheep.

In general, we obtained high validation rates for both SNPs (95.69%) and CNVs (76.19%). These results provided confidence that the pipeline applied for variant detection resolved a high quality variome in this study.

2. Functional enrichment of common and unique CNV regions among wild and domestic sheep

For the 6,929 common CNV regions that were present in all the 248 individuals of wild and domestic sheep, we used Gene Ontology (GO) and Kyoto Encyclopedia of Genes and Genomes (KEGG) pathway analyses to reveal the top 15 enriched GO terms. The biological process detected was associated with arginine metabolic process. The eight molecular functions were related to extracellular ligand-gated ion channel activity, ion channel activity, substrate-specific channel activity, channel activity and passive transmembrane transporter activity as well as calcium ion binding, oxygen

binding and heme binding (Supplementary Data 10). Moreover, we characterized 15 significant KEGG pathways (*P*-value <0.05), including metabolism pathways (e.g., Retinol metabolism, Insulin secretion, Pentose and glucuronate interconversions, N-Glycan biosynthesis, Ascorbate and aldarate metabolism, Purine metabolism and Starch and sucrose metabolism) and the Axon guidance and Circadian entrainment pathways, which function in neural system (Supplementary Data 10).

In addition, we revealed various functional categories between domestic sheep and Asiatic mouflon in the KEGG pathway and GO term analyses. These functional categories in domestic sheep (Supplementary Data 10) could reflect changes in body patterning, neurological processes (e.g., tameness), reproduction and immunity that have arisen during domestication and/or improvement. Nevertheless, different functional categories associated with unique CNV regions in Asiatic mouflon (Supplementary Data 10) suggested that selection pressures imposed by wild environments, which were different from those for farmed animals, had an important impact on their genomes.

3. Linkage disequilibrium (LD), effective population size (N_e), PSMC and genomic differentiation

We found differences in the LD estimates between Asiatic mouflon and domestic sheep (Fig. 1d, Supplementary Table 3). These values for domestic sheep, while being much higher than Asiatic mouflon and indicative of their low effective population size

(N_e), were nevertheless lower than those reported for dogs (344–834 kb)[15], albeit higher than the values for South African goats (< 10 kb)[16]. We found that Asiatic mouflon clearly exhibited lower levels of LD than domestic sheep (Supplementary Fig. 6a, b). Yak[17] and dog[18] displayed a consistent pattern of reduced LDs in wild populations relative to domesticates, which was most likely due to decreased effective sizes because of domestication bottlenecks and/or epistasis engendered by subsequent breeding selection[17,18]. However, similar patterns of demographic history were revealed between Asiatic mouflon and domestic sheep by the PSMC analysis (Supplementary Fig. 7). This finding confirmed previous reports, which found similar trends for domestic sheep and have suggested that glacial cycles were the main factors[14] influencing changes in population size.

For individual breeds, we observed substantial variations in the LDs, with a range from 8.2 kb in Bashibai to 68.3 kb in Ouessant (Supplementary Fig. 6 and Supplementary Table 3). This was possibly owing to differential effects of demographic history, artificial and natural selection, effective population size and/or other forces. One potential caveat was the slightly smaller sample sizes for the populations of Ouessant (n = 7), Drenthe Heathen (n = 5) and Shetland (n = 7), which might partly contribute to the relatively larger LDs estimated. Furthermore, we noted that only one African population (i.e., Dorper) was included in the comparison.

We observed a slightly higher level of interspecific differentiation between Asiatic

mouflon and domestic sheep (Asiatic mouflon vs. landraces: F_{ST} = 0.125; Asiatic mouflon vs improved breeds: F_{ST} = 0.132) than a previous estimate (F_{ST} = 0.093)[19]. Also, we detected genomic differences among domestic breeds of different geographic origins. Compared with other breeds, European sheep showed higher genomic similarities to Asiatic mouflon (Supplementary Fig. 5a). This may be explained by the dispersal of fat-tailed sheep, which replaced the more direct descendants of the wild ancestor[20,21]. Since the dispersal of fat-tailed sheep occurred as a separate event after the first introduction of domestic sheep[21-23] and they spread through only geographically restricted regions of Europe (e.g., Greece, Caucasus and via African introgression into the Italian Laticauda and Barbaresca), the vast majority of modern European breeds appeared to be the closest remaining relatives of the Asiatic mouflon. European breeds showed a higher level of LD (25.1 kb) than Asian populations (9.8 kb). This might be due to more intensive artificial selection in the European than Asian breeds and/or more heterogenous origins of the Asian breeds[24,25].

4. Putatively selected genes associated with domestication based on SNPs and CNVs

Of the 261 putatively genes revealed by two (XP-CLR and π ratio) or three (XP-CLR, π ratio and *iHS* or HKA) methods based on the SNPs, 14 genes have been associated with known functions in sheep in previous studies including *TET2*, *KDR*, *FLT1* and *HTRA1* for female reproductive traits (e.g., preimplantation development, oestrous cycle, litter size and off-season reproduction)[26-28], *GAK*, *CPLX1* and *PCGF3* for

recombination rate[29], *LEF1* for bone formation[30], *SLC11A1* for resistance to infection[31], *CAMK4* for steroidogenesis[32], *HOXA11* and *CTBP1* for fat deposition[33,34], *BCO2* for yellow-fat[35] and *CHGA* for photoperiod[36] (Supplementary Data 21). Among the other 22 genes previously found to be influenced by selection in other animals, *IGF2BP2* was shown to be associated with growth-related process in pig[37], *RFX3* and *HERC5* to be involved in immune function in cattle[38,39], *EDN3* to be implicated in pigmentation in a variety of mammals[40], *KITLG* and *RALY* to be associated with coat color in goat[41,42], pig[43] and dog[44], *PDE6B* to be related to photoreceptor development in chicken[45], *GTF2I* and *GTF2IRD1* to be relevant to behavior in dog[46], *MRPL41*, *MAN2B2* and *FGFRL1* to be responsible for reproductive traits in cattle and pig (e.g., follicle development, ovulation rate, conceptus development)[47-49] (Supplementary Data 21).

We noted that the most plausible domestication genes presented above were located within or near known QTL regions, indicating their roles to be involved in quantitative traits (Supplementary Data 18 and 19). However, the previously reported functions for most of the genes seemed to be unrelated to the quantitative traits (Supplementary Data 21). This could imply pleiotropic effects of the domestication genes, whose mutations potentially affect both fitness and production traits.

We also examined SVs and their frequencies in the 59 most plausible domestication genes (Supplementary Table 6 and Supplementary Data 21) across Asiatic mouflon

and domestic sheep populations. We found divergent frequency distribution for seven deletions (overlapping with *RFX3, AGMO, BCO2, LOC101112255, ADAMTSL3* and *SGCZ*) and three translocations (overlapping with *GTF2I, CAMK4* and *SGCZ*) between Asiatic mouflon and domestic sheep (Supplementary Table 9).

To explore the impact of CNVs on domestication, we carried out a selective sweep analysis of CNVs associated with domestication ((Drenthe Heathen in Europe, Hu sheep in East Asia, Altay sheep in Central Asia, Djallonké sheep in Africa and Karakul sheep in the Middle East) versus Asiatic mouflon) using V_{ST}[50]. Based on the top 1% V_{ST} value cutoff, we identified 137 putatively selected CNVs associated with domestication (Supplementary Data 23). After annotation, we found some of them to be overlapped with 30 functional genes (Supplementary Table 8), of which two CNVs (chr_6: 39,798,401–39,799,100 and chr_7: 57,751,201–57,751,490) were located within genes *SLIT2* and *FGF7*, to be functionally related to follicular development and fertility[51,52], one CNV (chr_2: 72,841,608–72,842,216) was located within *JAK2* to be associated with milk production traits[53], and six CNVs (chr_13: 9,710,901–9,711,101, 9,711,101–9,711,201, 9,711,201–97,12,400, 9,712,400–9,712,600, 9,712,600–9,712,700 and 9,712,700–9,712,800) were located within *KIF16B* to be involved in wool production traits[54], and one CNV each was located within *TCF7L1* (chr_3: 56,725,801–56,726,467), *PDE10A* (chr_8: 87,235,000–87,235,169) and *BCO2* (chr_15: 21,910,101–21,910,500) to be involved

in adipogenesis[35,55,56]. *PDE10A* was also found to be responsible for increased spleen size, oxygenated red blood cells and consequently high tolerance of hypoxia[57].

5. Selective signatures during breeding and improvement

Comparison of allele frequencies at non-synonymous SNPs in putatively selected genes revealed four of them (e.g., *SPAG8*, *FAM184B*, *PDE6B* and *PDGFD*) with significantly differentiated allele frequencies among domestic sheep breeds (Supplementary Data 29), implying their potential roles during breeding and improvement. For example, *SPAG8* was demonstrated to be functionally associated with growth rate in sheep[58] and the frequency of its variant allele (T) at a non-synonymous SNP G/T (chr_2: 52,480,881) was significantly (Mann-Whitney, P = 0.006) higher in two large-eared breeds (DLS and WGR) and one large-body-sized meat breed (SFK) than in the other sheep breeds. In addition, *FAM184B* was previously reported to be related to reproduction in chicken[59] and gait and conformation in horse[60], and its two non-synonymous SNPs (chr_6: 37,070,391 and 37,073,418) had significant differences in allele frequencies (Mann-Whitney, P = 0.018 for both SNPs) between the two breeds selected for meat production (SFK and DPS) and the other sheep breeds, suggesting its functional pleiotropy in multiple species and traits. Moreover, *PDE6B* was previously shown to be involved in photoreceptor development in chicken[45] and its variant allele frequencies of two non-synonymous mutations (chr_6: 116,759,053 and 116,785,562) were significantly different between Ouessant sheep and the other sheep breeds.

6. Selective signatures associated with phenotypic traits based on SNPs

We implemented genome-wide selection tests between domestic sheep breeds representing contrasting phenotypes for several traits (Supplementary Table 12). For reproduction, because a variety of genes and different genetic mechanisms have been identified in prolific sheep breeds[28], we performed separate pairwise-population selection tests between five prolific breeds (Finnsheep (FIN), Small-tailed Han sheep (SXW), Hu sheep (HUS), Gotland sheep (GOT) and Wadi sheep (WDS)) and one non-prolific breed, the Tan sheep (TAN), which produces a single lamb per year. We selected regions with differences in allele frequencies by XP-CLR (Supplementary Data 31 and Supplementary Figs. 12 and 13) and reduced π values (Supplementary Data 32) in the prolific breeds, and detected 113, 96, 89, 141 and 47 common selective sweeps (Supplementary Data 33). Among these, we found 17, 28, 36, 20 and 15 (Supplementary Data 34) and 7, 13, 6, 19 and one (Supplementary Data 35) to be overlapped with the selective signals detected by the *iHS* analysis (Supplementary Data 36) and the HKA test (Supplementary Data 37), respectively. Of the sweeps identified, we focused on fecundity-related genes involved in prolificacy and annotated 52 functional genes with high credibility (Supplementary Data 38), including some previously reported (e.g., *PTX3*, *BMP4*, *BMPR1B*, *PTGER4* and *SPP1*) and some novel (e.g., *UBE2Q1*, *CHRNB2*, *ADAR*, *TDRD10*, *KCNN3*, *IL6R*, *TOX*, *RXFP2*, *HIP1* and *POU2F1*) genes.

Similarly, we found many selected regions and novel functional genes, which may be responsible for other production traits, such as milk yield, wool fineness as well as meat production and growth rate (Supplementary Data 38 and Supplementary Figs. 14–16). More specifically, we identified functional genes *ALDH9A1*, *PDE4B*, *SND1*, *ARHGEF4*, *IQGAP3*, *ATF6* and *SLC40A1* to be associated with milk yield, fat and protein percentages (Supplementary Figs. 12 and 14 and Supplementary Data 38), *FGF6*, *KRT71*, *KRT74*, *KRT72*, *DSG2*, *LRRC15* and *IRF2BP2* to be related to wool fineness (e.g., coarse, fine, semi-fine and super-fine) (Supplementary Figs. 12 and 15 and Supplementary Data 38), and *PCCB*, *CSRP2*, *PHLPP1*, *CAT*, *LMO7* and *LMBR1* to be relevant to body configuration, meat production and growth rate (e.g., meat breed versus non-meat breed) (Supplementary Fig. 16 and Supplementary Data 38).

In addition to production traits, we also revealed selected regions and genes associated with morphological traits such as number of horns (polled, two-horned and polycerate: *RXFP2*, *HOXD1*, *HOXD3*, *HOXD10*, *TMEM45A* and *MEGF8*; Fig. 5, Supplementary Fig. 17 and Supplementary Data 38), pigmentation (black versus white: *RYR1*, *MITF*, *KIT*, *PDZK1*, *MLPH*, *NOTCH2*, *HPGDS* and *TBX2*; Supplementary Fig. 18 and Supplementary Data 38), number of nipples (two versus four nipples: *NRIP1*, *DAB1*, *FGF7*, *SYNDIG1L*, *VRTN*, *GRB10*, *DICER1* and *CTBP1*; Supplementary Fig. 19 and Supplementary Data 38) and ear size (large versus small ears: *BCL2L11*, *SLC26A4*, *EPHA6*, *PAX5*, *LEMD3* and *MSRB3*; Supplementary Figs. 12 and 20 and Supplementary Data 38).

To further dissect the genomic architectures underlying different phenotypic traits, we calculated the frequencies of the variant alleles at non-synonymous SNPs located up to 3-kb upstream and downstream of the selected genes (Figs. 4 and 5, Supplementary Figs. 10 and 12 and Supplementary Data 39). We identified divergence of variant allele frequency among breeds for the traits of reproduction, milk production, number of horns, wool fineness, ear size, number of nipples and coat color. For reproduction trait, we found two non-synonymous SNPs upstream of *BMPR1B*, which was considered as a major gene associated with fecundity[61]. The variant alleles attained high frequencies (90% and 80%) in the prolific Finnsheep but have much lower frequencies (mostly 10%–40%) in the non-prolific sheep (e.g., Large-tailed Han sheep (HDW), Tan sheep (TAN) and Duolang sheep (DLS)) and were unexpectedly absent in the prolific Hu sheep (Supplementary Figs. 10 and 12 and Supplementary Data 39). Conversely, the variant allele of another non-synonymous SNP downstream of *BMPR1B* was almost fixed (95%) in Hu sheep compared to the other sheep breeds (mostly below 50%) and the genotype pattern of *BMPR1B* in Hu sheep was significantly different (Mann-Whitney, $P < 0.001$) from those of other sheep breeds (Supplementary Fig. 12a,b and Supplementary Data 39). This implied that different molecular mechanisms underlay the prolificacy traits in different prolific sheep breeds (e.g., Finnsheep and Hu sheep), providing additional evidence for previously reported mechanisms responsible for the prolificacy traits in sheep[28].

For milk production, we found that the variant allele frequencies of 15 non-synonymous SNPs in *ARHGEF4*, a candidate gene for milk production in cattle[62], were dramatically reduced in high-yield breeds (e.g., East Friesian Dairy sheep (EFR)) than in low-yield breeds (e.g., Finnsheep (FIN), Suffolk sheep (SFK) and Dorper sheep (DPS)) (Supplementary Figs. 10 and 12g,h and Supplementary Data 39). Also, we observed significantly different genotype patterns of *ARHGEF4* (Mann-Whitney, P = 0.004) and *IQGAP3* (Mann-Whitney, P = 0.024) between high- and low-yielding sheep (Supplementary Figs. 12g,h and 14). The amino acid substitutions (e.g., valine/leucine, serine/proline and glycine/alanine; Supplementary Data 39) induced by the variant alleles in *ARHGEF4* may therefore have a negative relationship with sheep milk yields.

For two horn-related genes (*HOXD1* and *RXFP2*)[63,64], we observed that the variant allele frequency of one non-synonymous SNP locus downstream of *HOXD1* was greatly elevated in the polycerate sheep breed (e.g., Sishui Fur Sheep (SSS)) than in the other sheep breeds (e.g., SXW, TAN and HUS), and the genotype patterns of *HOXD3* and *HOXD8* in the polycerate breed (e.g., SSS) were distinct (Mann-Whitney, P = 0.022) from two-horned breeds (e.g., SXW, TAN and DRS) (Fig. 5d, f and Supplementary Data 39). Also, the variant alleles of two non-synonymous SNPs downstream of *RXFP2* exhibited significantly higher frequencies (chr_10: 29432742, Mann-Whitney, P = 0.005; chr_10: 29432846, Mann-Whitney, P = 0.004) in four horned breeds (e.g., SSS, SXW, TAN and DRS; 80%–100%) than in polled breeds

(e.g., 0% in Hu sheep) (Fig. 5e and Supplementary Data 39), and the genotype pattern of *RXFP2* in the horned breeds was distinct (Mann-Whitney, *P* = 0.012) from the polled breeds (Fig. 5g). These findings suggested that the variant alleles in *HOXD1* and *RXFP2* were the causal mutations determining the horn conditions in sheep.

Similarly, we detected significant differences in variant allele frequencies of non-synonymous SNPs and distinct genotype patterns in genes for other traits among sheep breeds (Supplementary Fig. 10 and Supplementary Data 39). For wool fineness, we found different frequencies for variant alleles at *KRT74* and *KRT72* and different genotype pattern (Mann-Whitney, *P* < 0.001) for the region encompassing *KRT1*, *KRT2*, *KRT71*, *KRT72* and *KRT 74* between fine-wool (e.g., SHE, MFW and MSF) and coarse-wool breeds (e.g., ALS and TAN) (Supplementary Fig. 12e,f). For ear size, we identified different patterns for variant alleles at *LEMD3*, *MSRB3* and *TSHR* and for genomic regions in *LEMD3* (Mann-Whitney, *P* = 0.083) and *MSRB3* (Mann-Whitney, *P* = 0.030) between the breeds with large ears (e.g., WGR and DLS) and those with small ears (e.g., SHE) (Supplementary Fig. 12c.d). For number of nipples, we found different frequencies for one variant allele at *NRIP1* and significantly different genotype pattern (Mann-Whitney, *P* = 0.002) for *SYNDIG1L* between the breed with four nipples (e.g., WDS) and breeds with two nipples (e.g., DPS and SFK) (Supplementary Fig. 19). Additionally, we observed different genotype pattern for *MITF* between black headed Dorper sheep (e.g., BDP) and white headed Dorper sheep (e.g., WDP)/white coat sheep (e.g., HUS) (Mann-Whitney, *P* = 0.001)

or dark coat sheep (e.g., CLS, GOT, SOL) (Mann-Whitney, $P = 0.017$) (Supplementary Fig. 18). Importantly, we found that amino acids changes in *KRT74* (histidine/asparagine) and *KRT72* (isoleucine/valine, glutanine/arginine and alanine/valine) induced by the variant alleles could influence characteristics of wool[65], yielding further insights into the molecular basis for wool fineness in sheep.

7. Selective signatures associated with phenotypic traits based on CNVs

In addition to SNP variants, CNVs have increasingly been focused upon to explore genes associated with complex traits in domestic animals including sheep[66]. Herein we presented a selective sweep analysis of CNVs for 12 phenotypic traits (43 pairwise comparisons between domestic sheep breeds; Supplementary Table 12) using V_{ST}[50]. Using the top 1% of the V_{ST} values, we identified an average of 137 trait-associated CNVs for each pairwise comparison (Supplementary Data 41), which could be presented as part of the selective signatures. We annotated the putatively selected CNVs and discovered 61 trait-associated CNVs to be overlapped with 50 functional genes, which were known to be responsible for fat tail, reproduction, milk related traits, wool related traits, number pf nipples, horn, body size, ear size and coat color (Fig. 4b, c Supplementary Data 42 and Supplementary Figs. 9 and 13–20). Particularly, we found the presences of both selected CNVs and SNPs in genes *FGF7* and *CNOT3* responsible for tail type, *SENP6* for reproduction and *ATM* and *LINGO2* for body size.

8. GWAS analyses based on CNVs

We also implemented GWAS analyses using the CNV data for litter size, numbers of horns and nipples. We detected a total of 11 significant association signals for the three traits with the thresholds $-\log_{10}$(0.05/total CNVs) ≥ 5.286, 5.294 and $-\log_{10}$(*P*-value) = 4, respectively (Fig. 5b and Supplementary Table 14). Specifically, we found one CNV located on chr_27: 106,688,516–106,690,665 close to *SMARCA1* gene and another CNV located on chr_1: 128,659,801–128,662,221 close to *APP* gene, both were found to be significantly associated with litter size (Supplementary Fig. 23 and Supplementary Table 14). The two genes were reported to be involved in regulation of RNA synthesis before reaching oocyte MII stage and associated with porcine embryos growth and development[67]. For number of horns, we identified one most significant CNV (chr_10: 29,433,062–29,434,913) to be overlapped with *RXFP2* (Fig. 5b and Supplementary Table 14) and also overlapped with previously reported QTL of horns on chromosome 10 (12,606,797–45,964,265) (permutation test, *P* = 0.003) (Supplementary Tables 5 and 15). For number of nipples, we found one significant CNV located on chr_10: 64,952,791–64,955,595 (Supplementary Table 14) close to *GPC5* gene, a member of the heparin sulfate proteoglycans (HSPGS) that was shown to have a distinct role in regulating the interaction between growth factor and receptor and also a crucial role in breast cancer[68].

9. Overlapping between detected signatures and known QTLs

Selective signatures associated with domestication based on CNVs. Of the 137

selected CNVs, 120 were coincident with known QTLs[69] (permutation test, $P < 0.001$) (Supplementary Table 5), including milk-, meat- and wool-related traits as well as traits for body and testis weights (Supplementary Table Data 24).

Selective signatures associated with phenotypic traits based on SNPs. We compared the 133 commonly selected regions associated with different phenotypes (e.g., production, morphological and physiological traits) by XP-CLR, π-ratio and *iHS* analyses as well as the 95 commonly selected regions by XP-CLR, π-ratio and HKA analyses with previously mapped QTLs. We found that 30 of the selective sweeps by XP-CLR, π-ratio and *iHS* and 43 of the selective sweeps by XP-CLR, π-ratio and HKA overlapped with known QTLs[69], which are associated with a number of traits such as such as reproduction, milk, growth, meat and carcass, fat deposition in the tails and wool fineness (Supplementary Table 5, Supplementary Data 43 and 44). Specifically, for XP-CLR, π-ratio and *iHS*, the number of QTLs (shorter than 100 Mb) overlapping for traits such as reproduction, milk, growth, meat and carcass, fat deposition in the tails are 3 (permutation test, $P < 0.01$), 1 (permutation test, $P < 0.05$), 20 (permutation test, $P < 0.001$), 7 (permutation test, $P < 0.05$), respectively (Supplementary Table 5 and Supplementary Data 43). For XP-CLR, π-ratio and HKA, the overlapping numbers of QTLs (shorter than 100 Mb) for the above traits are 1 (permutation test, $P < 0.001$), 4 (permutation test, $P < 0.001$), 32 (permutation test, $P < 0.001$) and 2 (permutation test, P < 0.05), respectively (Supplementary Table 5 and Supplementary Data 44).

Selective signatures associated with phenotypic traits based on CNVs. We found 782 trait-associated selected CNVs to be overlapped with known QTLs[69] (shorter than 100 Mb) for distinct traits, including 11 QTLs for tail fat deposition, six for reproduction, 38 for milk, 119 for growth, meat and carcass (permutation test, $P < 0.001$), 13 for wool, one for coat color (permutation test, $P = 0.007$) and one for horns (Supplementary Table 5 and Supplementary Data 45).

Overall, by comparing the selective sweeps with the known QTLs[69] associated with particular traits, we observed a large number of overlapping regions, providing strong confidence for the selective sweeps detected based on both SNPs and CNVs. More importantly, the identification of SNPs and CNVs in these previously described genes and QTLs provided a more detailed dissection of genomic architectures and generated novel insights into the molecular mechanisms underpinning sheep phenotypic traits. Through calculating the proportion of phenotypic variation explained by the genetic variants identified in GWAS, our results highlighted the importance of these SNP variants for the phenotypic diversity of the traits, allowing us to infer that for some traits (e.g., litter size and number of nipples), multiple loci with relatively small effects accounted for the phenotypic variance, whereas other traits (e.g., polled phenotype) may have derived from large effects of a few major loci.

Supplementary Figures

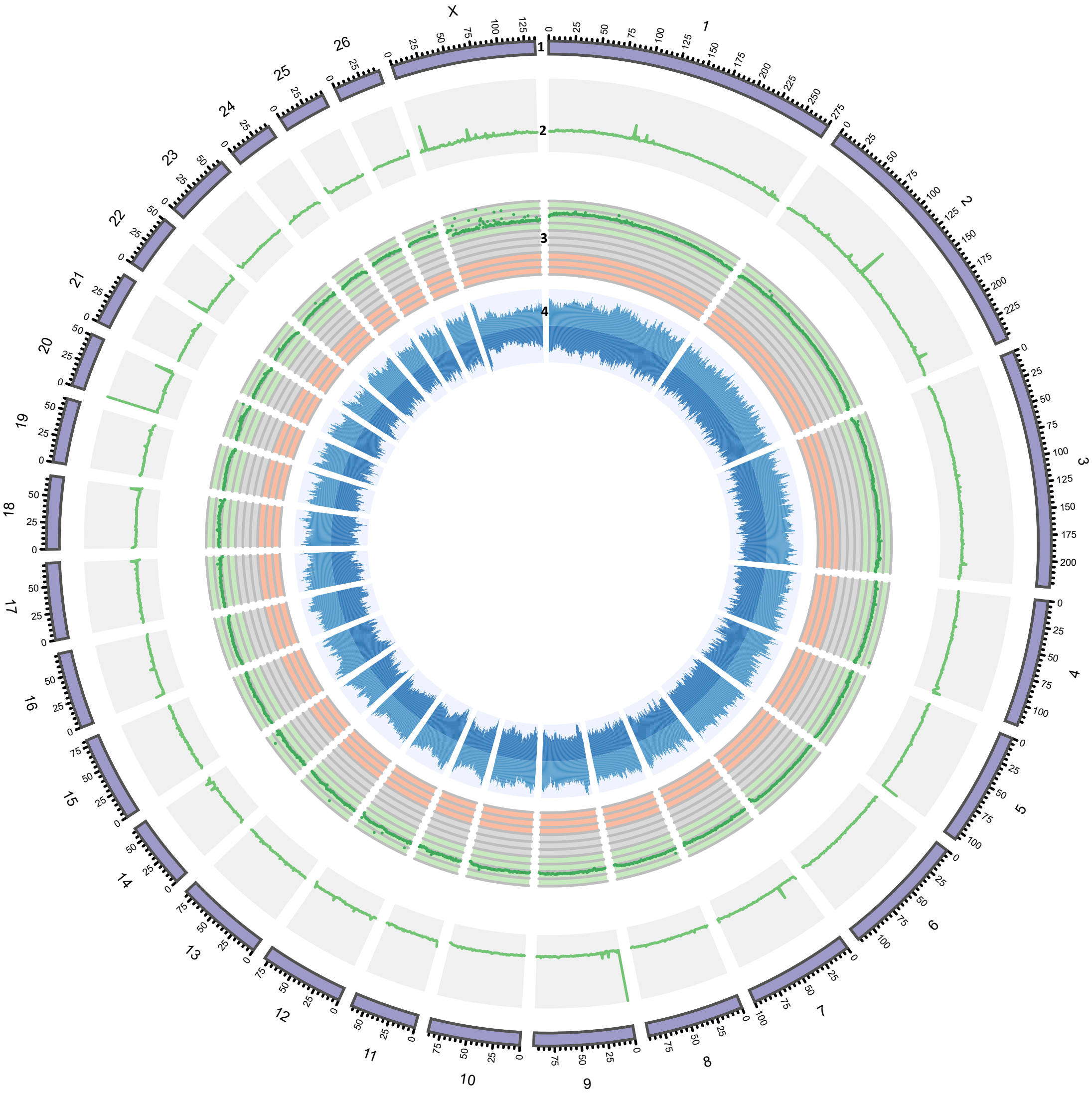

Supplementary Figure 1. A Circos image representing depth of sequencing and variations. (**1**) 27 chromosomes (1–26 and X) with scale of Mb genome. (**2**) Sequence coverage in 248 individuals (minimum: 5,309; maximum: 24,106). The range of coverage plot axis is 0 to 25,000. (**3**) Depth of SNPs regions in 248 individuals. The range of SNP axis is 0 to 3,060 (orange circle: 0–612; purple circle: 612–1,530; green circle: 1,530–3,060). (**4**) Depth of indels in 248 individuals (deletions, light blue, 14–2,292; insertions, dark blue, 10–1,518). Source data are

provided as a Source Data file.

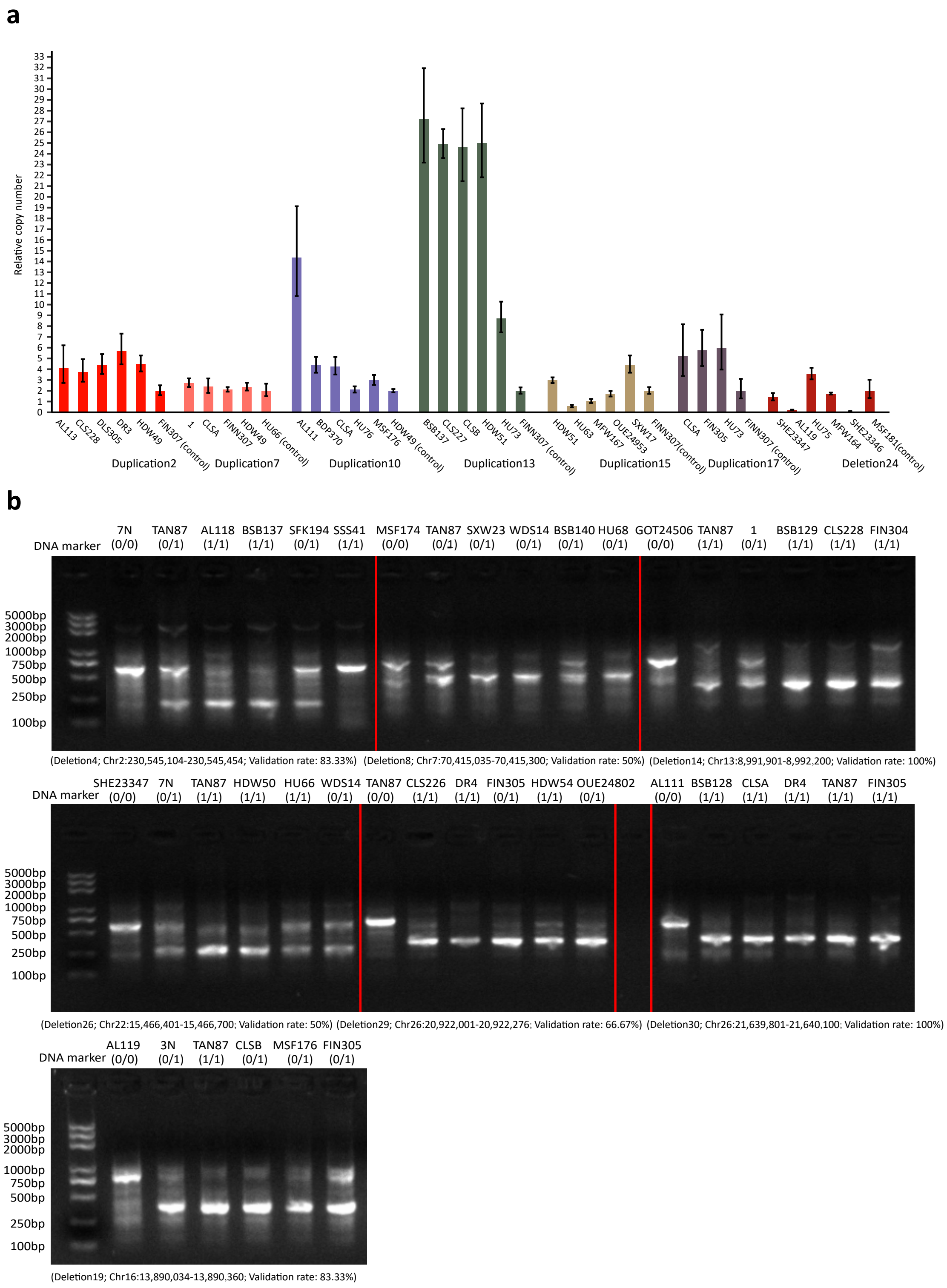

Supplementary Figure 2. Validation of CNVs by qPCR and regular PCR. (**a**) Relative quantification of six duplications and one deletion by qPCR. The x and y axis represent sample ID and relative CNVs expression level ($2\times2^{-(\Delta\Delta CT\pm SD)}$, n=3). (**b**)

Seven deletions genotyped by the DELLY calls and validated by regular PCR products checked through the lengths of the products by gel assays. The first sample from the left for each deletion is taken as the control sample. The genotype shown on top of each gel figure is the genotype for each deletion called by the DELLY v.0.7.9. Each PCR was repeated three times independently with similar results. Source data are provided as a Source Data file.

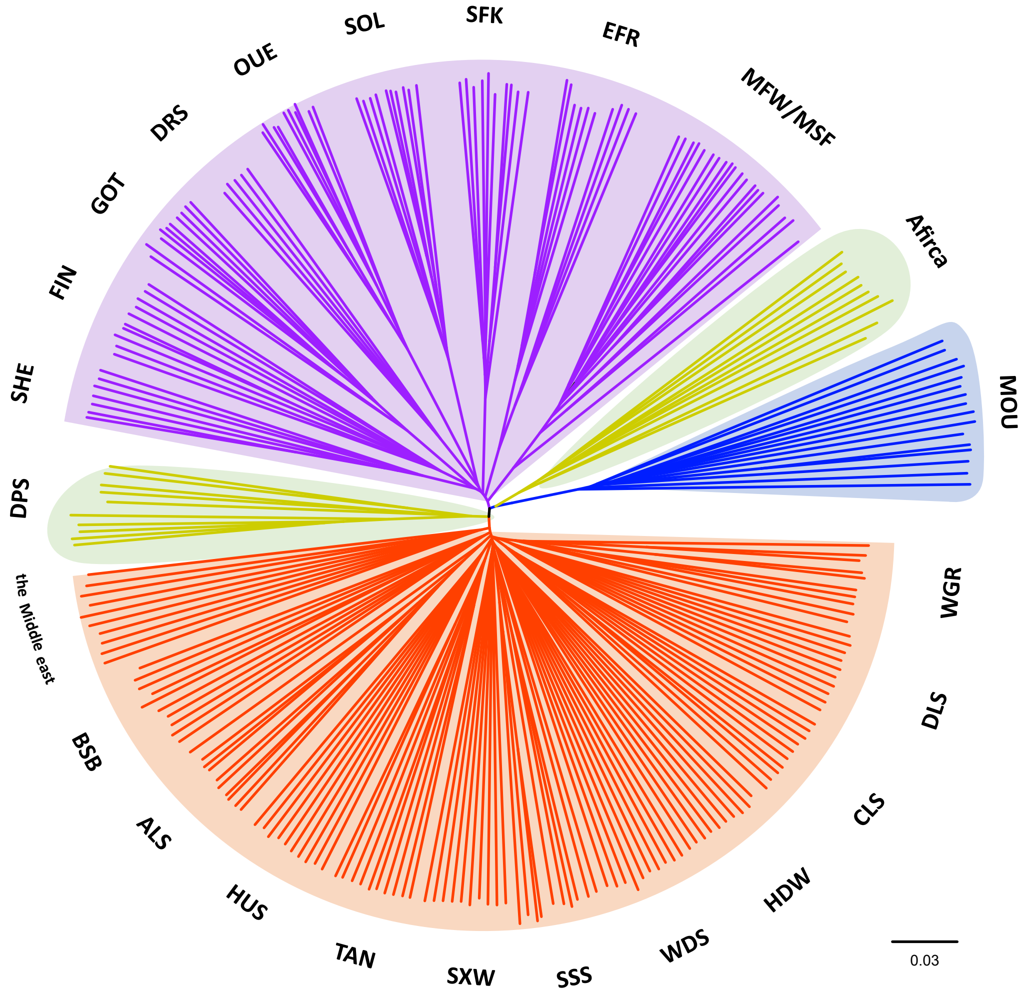

Supplementary Figure 3. Phylogenetic neighbor-joining tree of 248 individuals with Asiatic mouflon as an outgroup. The breed IDs are shown on the branches. Source data are provided as a Source Data file.

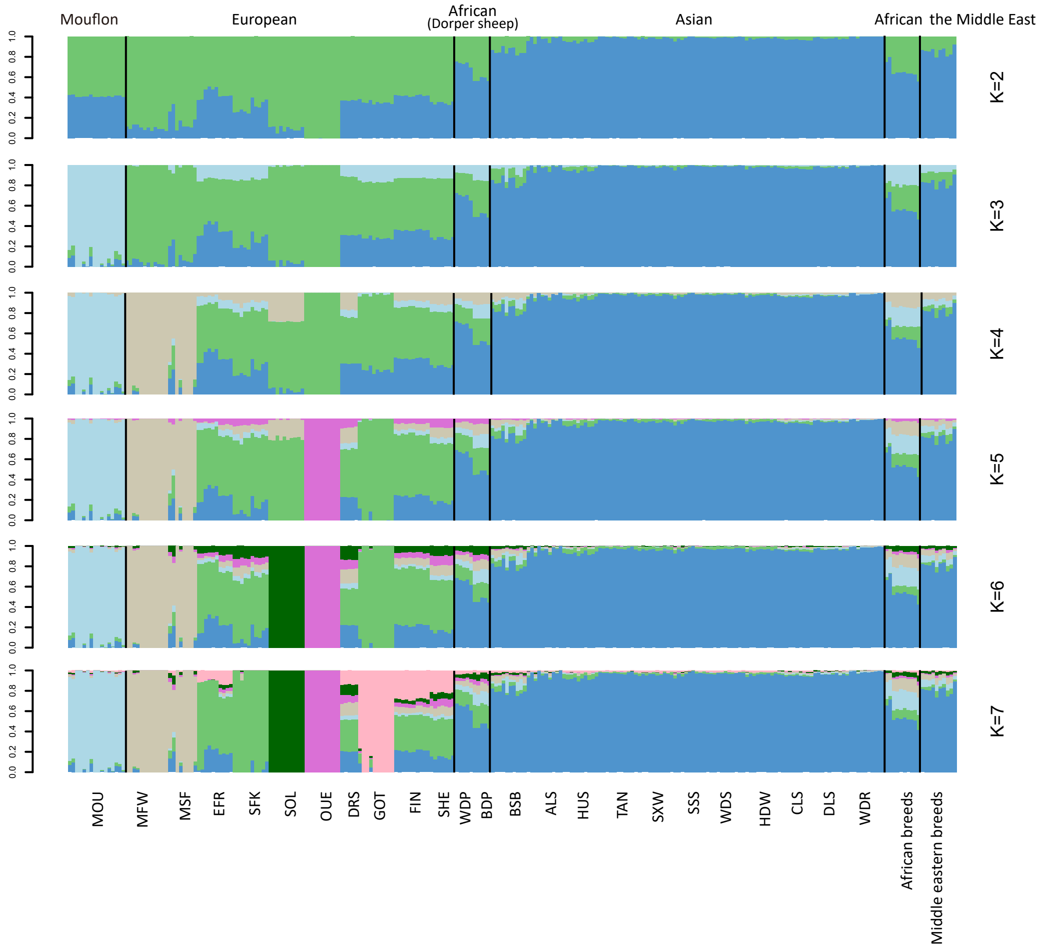

Supplementary Figure 4. Genetic structure analysis of 248 individuals from Asiatic mouflon and sub-groups of domestic sheep. The length of each colored segment in each bar represents the proportion contributed by ancestral populations (K = 2–7) for an individual. Source data are provided as a Source Data file.

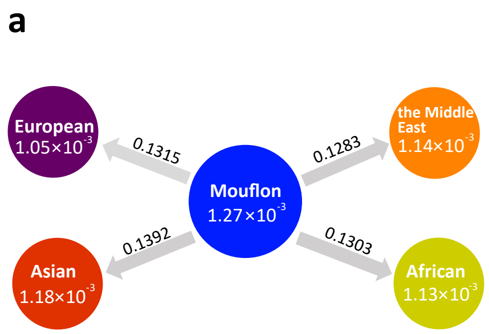

b

F_{ST}	Mouflon	European	the Middle East	Asian	African
Mouflon	0	0.1315	0.1283	0.1392	0.1303
European	0.1315	0	0.0479	0.0505	0.0528
the Middle East	0.1283	0.0479	0	0.0126	0.0370
Asian	0.1392	0.0505	0.0126	0	0.0459
African	0.1303	0.0528	0.0370	0.0459	0

P-value	Mouflon	European	the Middle East	Asian	African
Mouflon	0	0.0104	0.0280	0.0084	0.0160
European	0.0104	0	0.1746	0.0176	0.0766
the Middle East	0.0280	0.1746	0	0.3558	0.2541
Asian	0.0084	0.0176	0.3558	0	0.0819
African	0.0160	0.0766	0.2541	0.0819	0

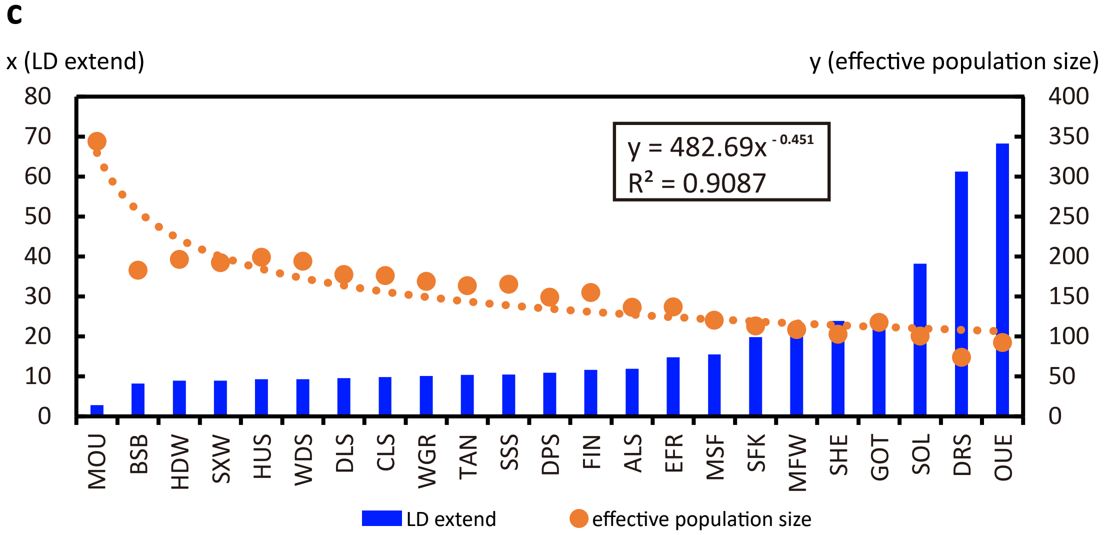

Supplementary Figure 5. Genetic diversity, pairwise F_{ST}, LD and the effective population size between Asiatic mouflon and sub-groups of domestic sheep. (**a**) Genomic diversity (π) and population differentiation in Asiatic mouflon and four domestic sheep sub-groups (the Middle East, European, African and Asian) based on the clades of the phylogenetic tree (Fig. 1b) with less than 10% missing data. (**b**) Pairwise F_{ST} and p values between Asiatic mouflon, the Middle East, European, African and Asian domestic sheep. (**c**) Scatter plots on LD (measured as r^2) and bar charts on the effective population sizes in Asiatic mouflon and 22 domestic sheep populations. The number for each population is shown in Supplementary Data 1. Source data are provided as a Source Data file.

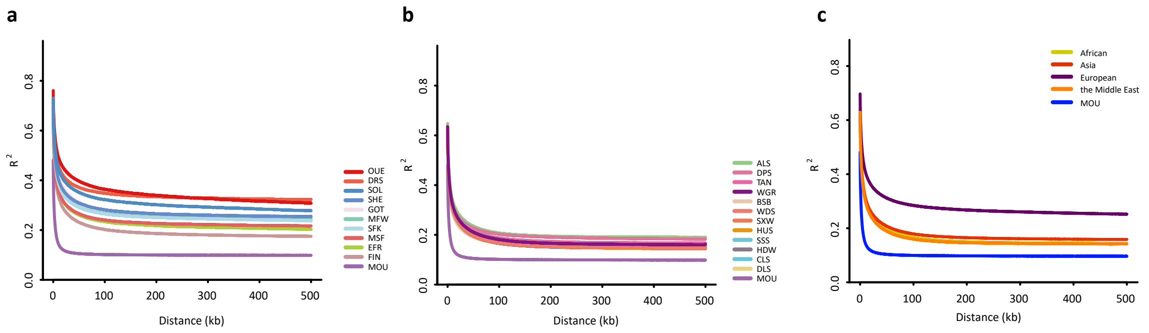

Supplementary Figure 6. Decay of linkage disequilibrium in (**a**) Asiatic mouflon and 10 different domestic sheep populations (European sheep); (**b**) Asiatic mouflon and 12 different domestic sheep populations (Asian and African sheep); (**c**) Asiatic mouflon and the four sub-groups of domestic sheep of different geographical origins. Source data are provided as a Source Data file.

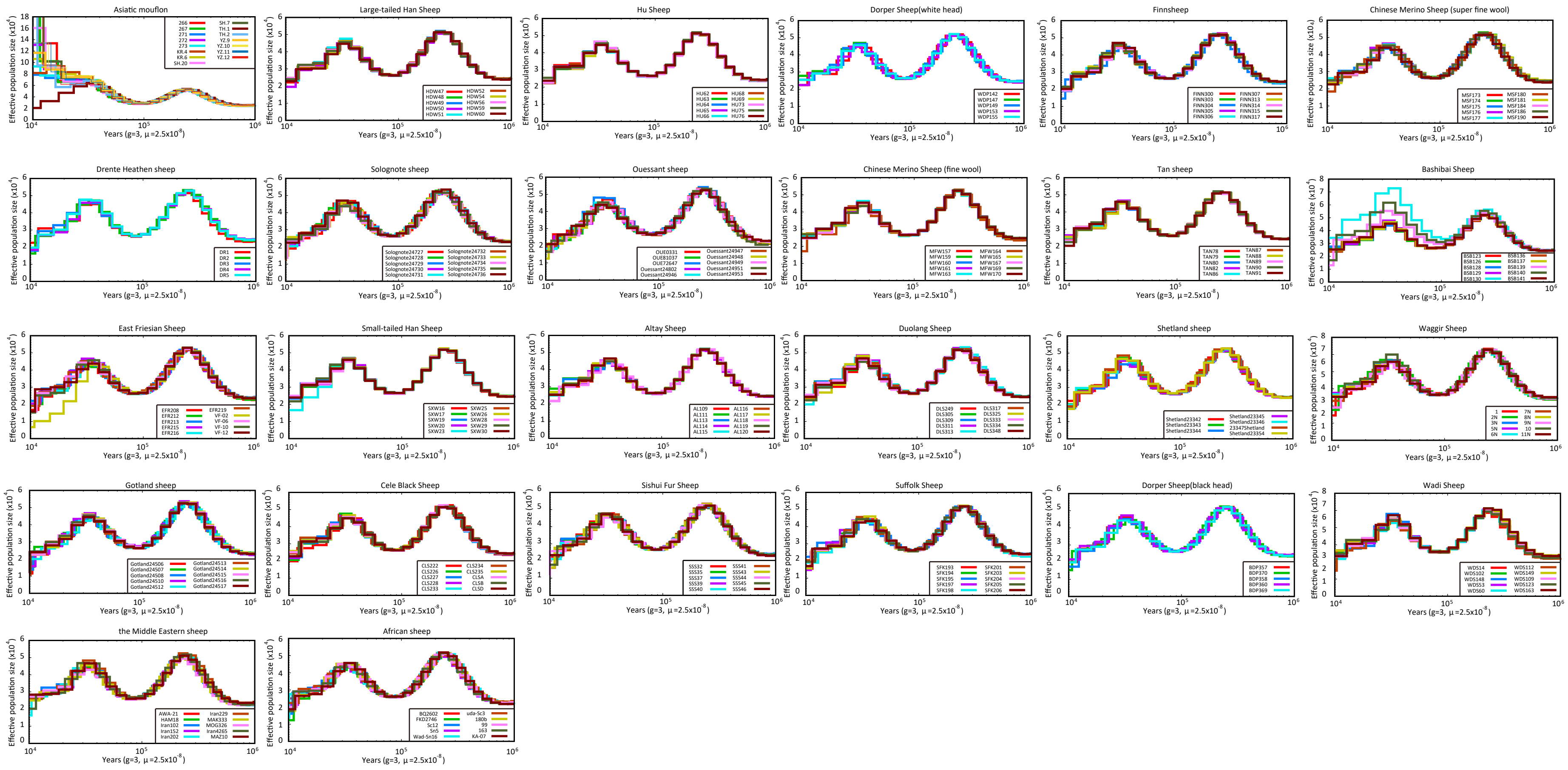

Supplementary Figure 7. Pairwise sequential Markovian coalescent (PSMC) analysis results. Asiatic mouflon and sub-groups of domestic sheep exhibit inferred variations in their effective population sizes (*N*e) over the last 10^6 years. Source data are provided as a Source Data file.

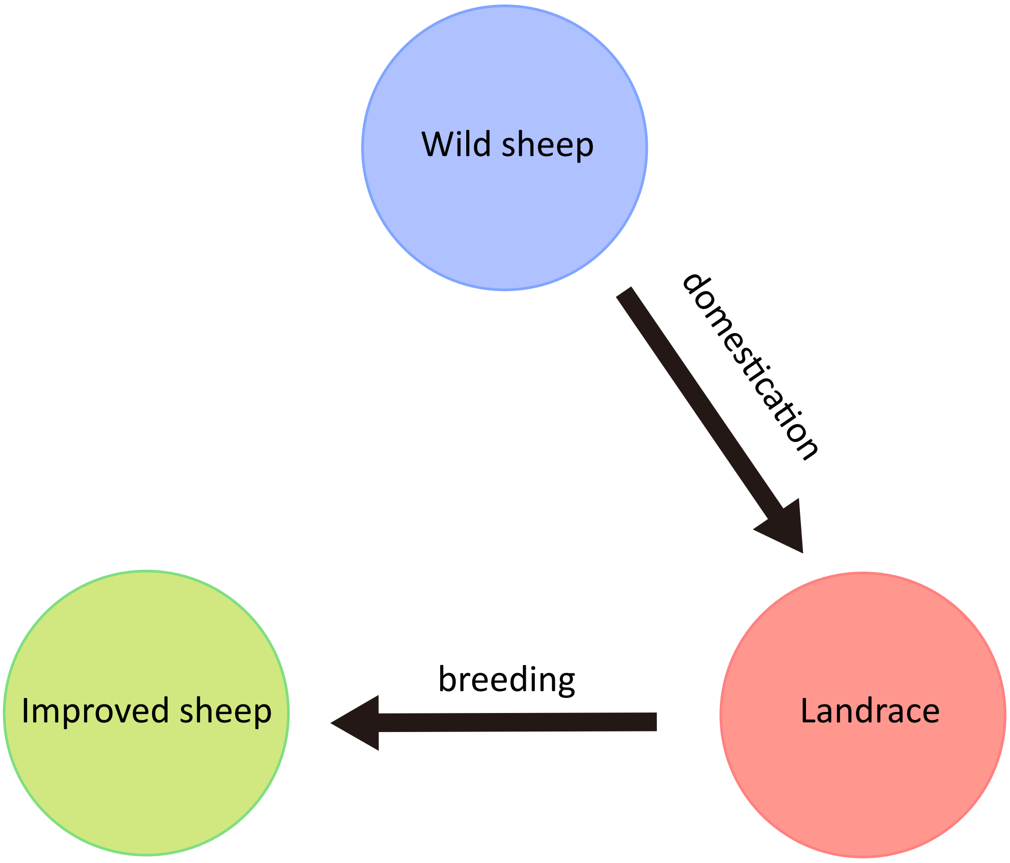

Supplementary Figure 8. Diagram illustrating the process of domestication and breeding in sheep.

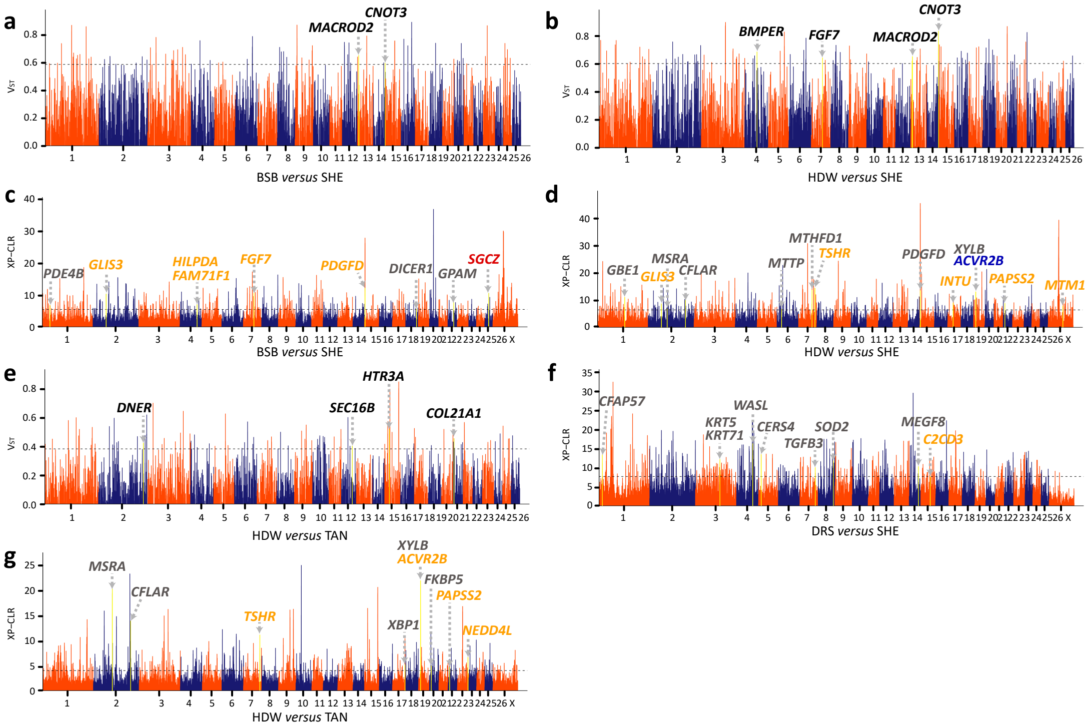

Supplementary Figure 9. Genome-wide screening and functional annotations of selected regions associated with tail type. Through comparing long-fat-tailed sheep (HDW), fat-rumped sheep (BSB) and long wooly tailed sheep (DRS) with short-fat-tailed sheep (TAN) and thin-tailed sheep (SHE), whole-genome copy number variations (CNVs) and SNPs data were used to perform selective analysis. (**a, b, e**) Statistic V_{ST} in these five comparisons is plotted against the position of CNVs on each of the 26 autosomes, and the thresholds are V_{ST} value ≥ 0.585 (a), 0.602 (b) and 0.383 (e). Candidate genes overlapping with significantly selected regions are marked by black color. (**c, d, f, g**) Manhattan plots of selective analysis by XP-CLR and the thresholds are XP-CLR ≥ 5.607 (c), 6.532 (d), 7.785 (f) and 4.143 (g). Candidate genes overlapped with regions which were significantly selected by XPCLR & ln(π ratio)/ln(2), XPCLR & ln(π ratio)/ln(2) & *iHS*, XPCLR & ln(π ratio)/ln(2) & HKA and XPCLR & ln(π ratio)/ln(2) & *iHS* & HKA are marked by grey, orange, blue and red colors respectively. Source data are provided as a Source Data file.

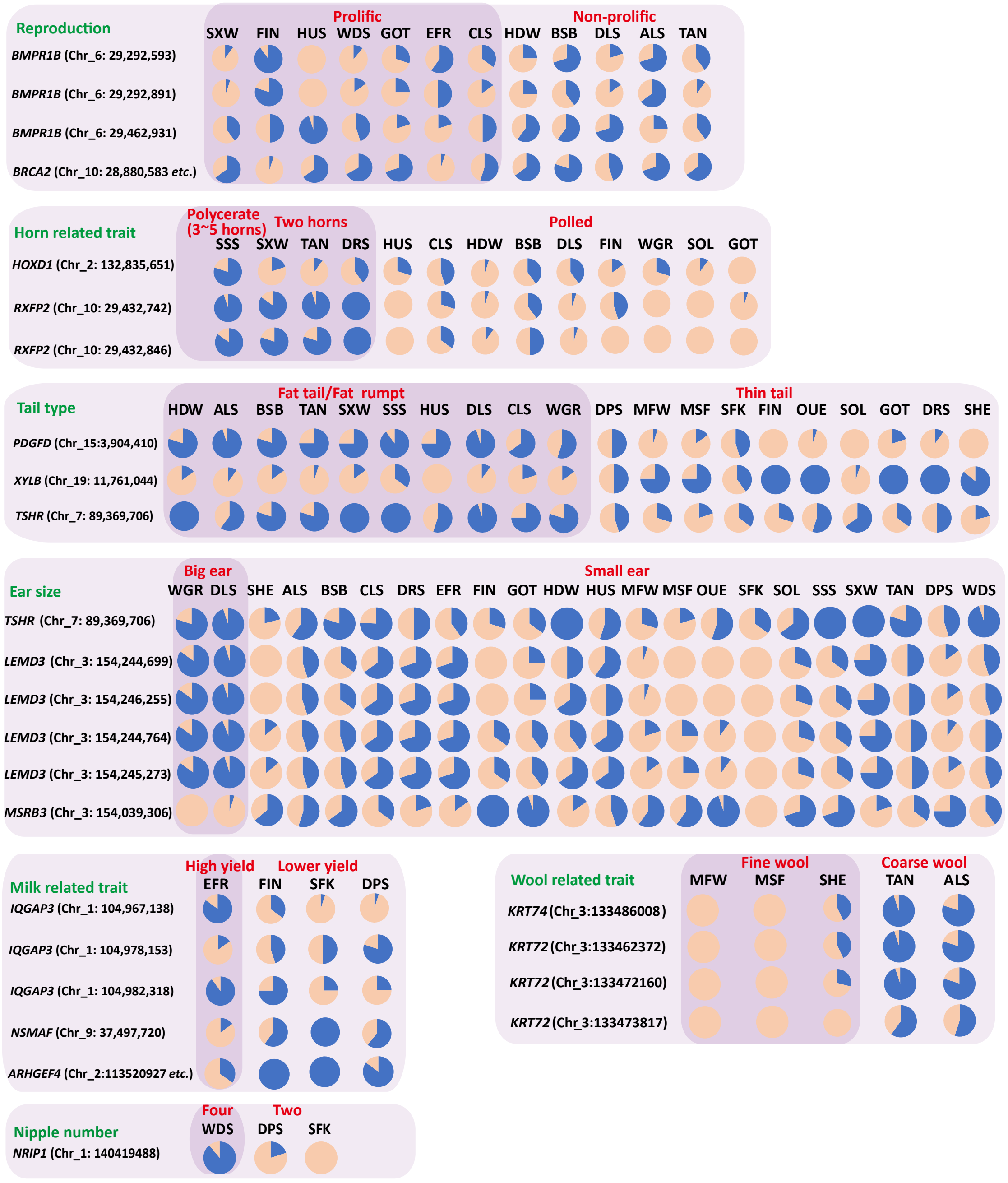

Supplementary Figure 10. Allele frequencies of non-synonymous SNPs and SNPs located at the upstream 3-kb and downstream 3-kb of candidate genes. These gene are associated with various phenotypic (e.g., production and morphological) traits in different domestic sheep breeds. Blue and pink indicate the variant and reference allele frequency, respectively.

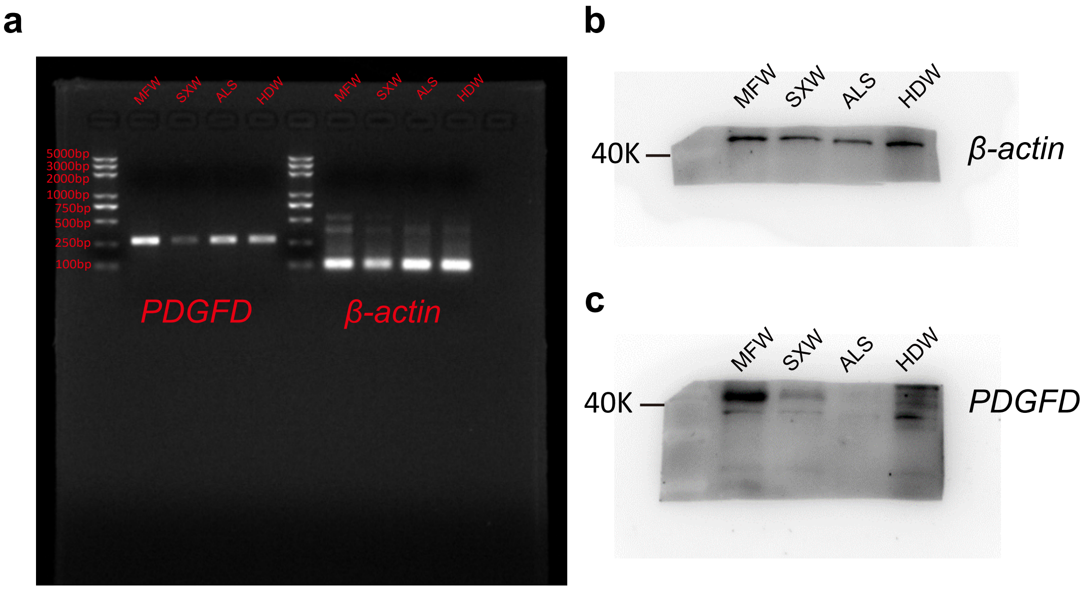

Supplementary Figure 11. Full scans for the RT-PCR and Western blot. (**a**) A full scan of the expression pattern of control gene *β-actin* (right) and target gene *PDGFD* (left) in tail fat examined by RT-PCR with the control sample (The thin-tailed Merino sheep; MFW) and target samples (long fat-tailed sheep (HDW), fat-rumped sheep (ALS) and short-fat-tailed sheep (SXW)). (**b, c**) The full scan of the expression pattern of control protein coding by *β-actin* and target protein coding by *PDGFD* examined by western blot with the control sample (MFW) and target samples (HDW, ALS and SXW), respectively. Samples derived from the same experiment and blots were processed in parallel. All experiments were repeated three times with similar results. Source data are provided as a Source Data file.

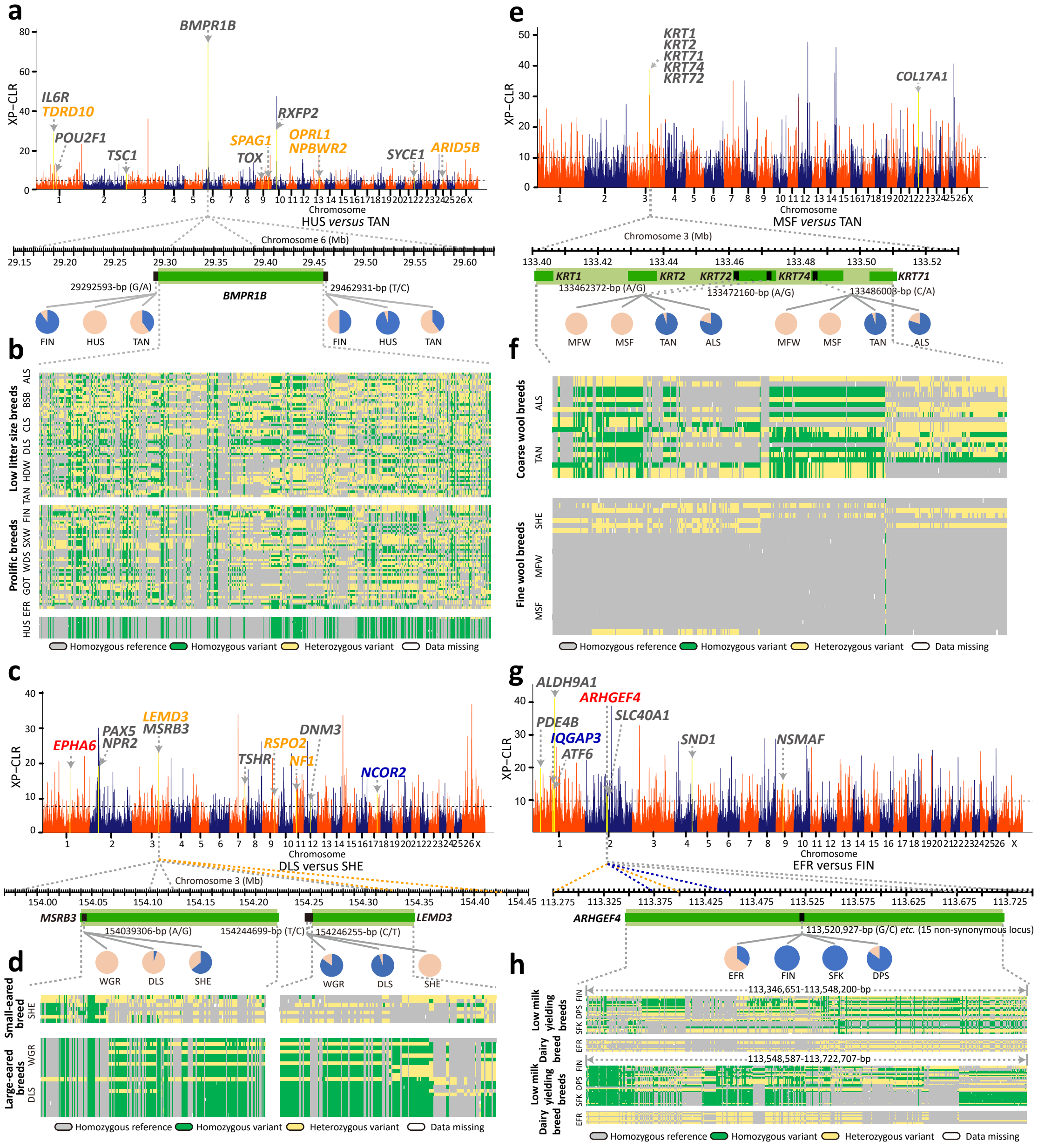

Supplementary Figure 12. Genome-wide annotations of selected regions associated with different phenotypic traits. (**a**) Manhattan plot for reproduction traits (HUS versus TAN), and the allele frequency distribution of two non-synonymous SNPs at *BMPR1B* in two prolific breeds (FIN and HUS) and one non-prolific breed (TAN). The horizontal dashed line corresponds to the genome-wide significance threshold (XP-CLR = 4.567). (**b**) Genotype patterns of *BMPR1B* among six prolific breeds and six low litter size breeds (**c**) Manhattan plot

for ear size (DLS versus SHE), and the allele frequency distribution of one non-synonymous SNP at *MSRB3* and two non-synonymous SNPs at *LEMD3* in two large-eared breeds (WGR and DLS) and one small-eared breed (SHE). The horizontal dashed line corresponds to the genome-wide significance threshold (XP-CLR = 7.690). (**d**) Genotype patterns of *MSRB3* and *LEMD3* among two large-eared breeds and one small eared breed. (**e**) Manhattan plot for wool fineness (MSF versus TAN), and the allele frequency distribution of two non-synonymous SNPs at *KRT72* and one non-synonymous SNP at *KRT74* in two fine wool sheep breeds (MFW and MSF) and two coarse wool sheep breeds (TAN and ALS). The horizontal dashed line corresponds to the genome-wide significance threshold (XP-CLR = 10.026). (**f**) Genotype patterns of *KRT1, KRT2, KRT72, KRT74* and *KRT71* among three fine wool breeds and two coarse wool breeds. (**g**) Manhattan plot for milk yielding (EFR versus FIN), and the allele frequency distribution of one non-synonymous SNP at *ARHGEF4* in dairy breed (EFR) and three low milk yielding breeds (FIN, SFK and DPS). The horizontal dashed line corresponds to the genome-wide significance threshold (XP-CLR = 9.723). (**h**) Genotype pattern of gene *ARHGEF4* among one dairy breed and three low milk yielding breeds. In the figures a, c, e, g, the bottom panel shows a zoomed-in view of focused gene (e.g., indicated by green boxes) and overlapped or nearby selective sweep region (e.g., the region between two oblique lines on the black axes). The pie charts represent the spectrum of allele frequencies at the non-synonymous locus of the focused genes in contrasting breeds. In the pie charts, the variant allele is indicated in blue while the reference allele in pink. In the

Manhattan plots, candidate genes overlapped with regions which were significantly selected by XPCLR & ln(π ratio)/ln(2), XPCLR & ln(π ratio)/ln(2) & *iHS*, XPCLR & ln(π ratio)/ln(2) & HKA and XPCLR & ln(π ratio)/ln(2) & *iHS* & HKA are marked by grey, orange, blue and red colors, respectively.

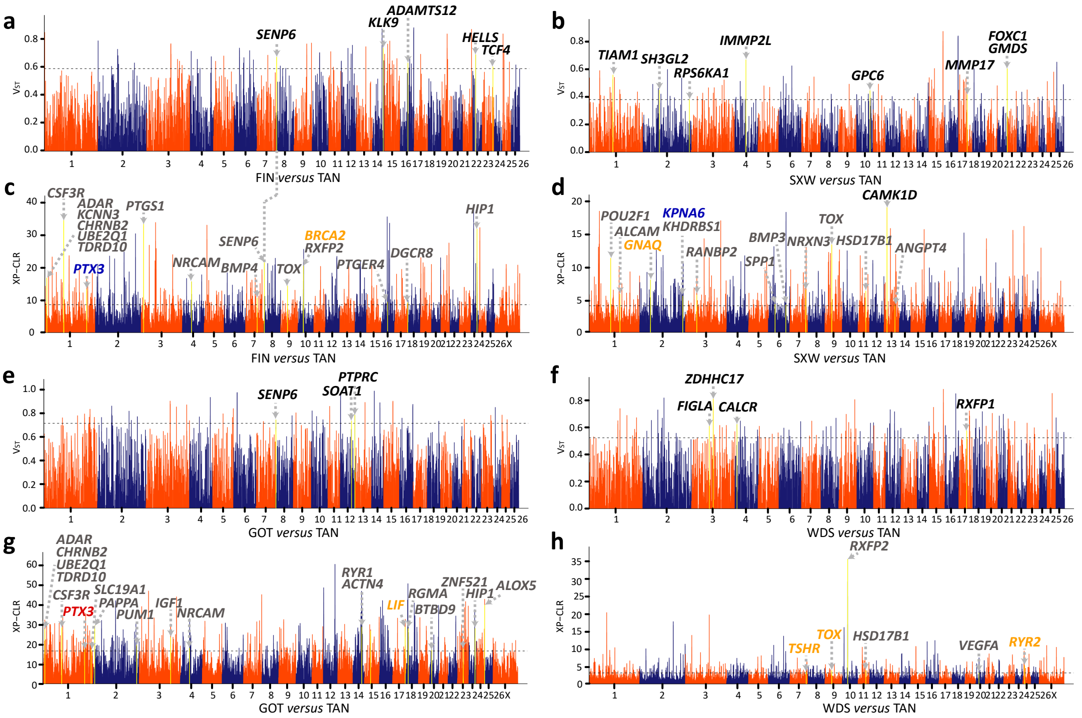

Supplementary Figure 13. Genome-wide screening and functional annotations of selected regions associated with reproduction trait. Whole-genome copy number variations (CNVs) and SNPs data were used to perform selective analysis through pairwise comparisons between five prolific breeds (FIN, HUS, GOT and WDS) and one non-prolific breed (TAN). (**a, b, e, f**) Statistic V_{ST} in the four comparisons is plotted against the position of CNVs on each of the 26 autosomes, and the thresholds are V_{ST} value $\geq$ 0.588 (a), 0.394 (b), 0.716 (e) and 0.522 (f). Candidate genes overlapped with significantly selected regions are marked by black color. (**c, d, g, h**) Manhattan plots of selective analysis by XP-CLR and the thresholds are XP-CLR $\geq$ 8.705 (c), 4.185 (d), 16.646 (g) and 3.306 (h). Candidate genes overlapped with regions which were significantly selected by XPCLR & ln(π ratio)/ln(2), XPCLR & ln(π ratio)/ln(2) & *iHS*, XPCLR & ln(π ratio)/ln(2) & HKA and XPCLR & ln(π ratio)/ln(2) & *iHS* & HKA are marked by grey, orange, blue and red colors, respectively. Source data are provided as a Source Data file.

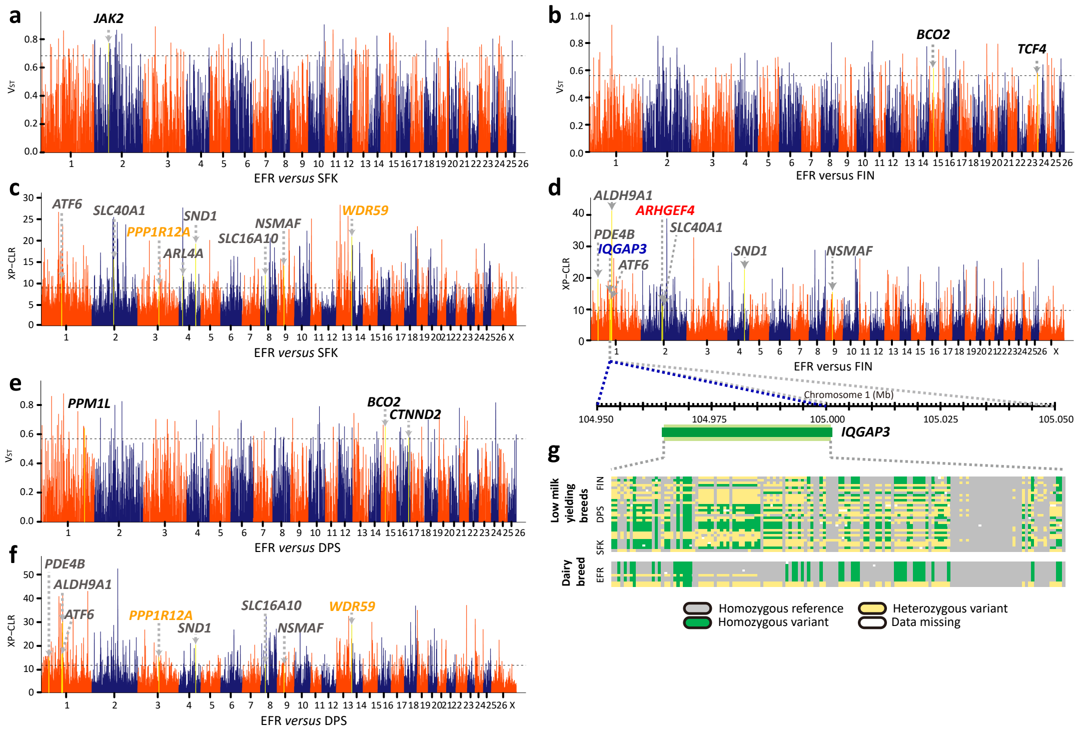

Supplementary Figure 14. Genome-wide screening and functional annotations of selected regions associated with milk production. Whole-genome copy number variations (CNVs) and SNPs data were used to perform selective analysis through pairwise comparisons between one improved breed (EFR, high milk yield) and three low milk yielding breeds (SFK, FIN and DPS). (**a, b, e**) Statistic V_{ST} in three comparisons is plotted against the position of CNVs on each of the 26 autosomes, and the thresholds are V_{ST} value ≥ 0.684 (a), 0.559 (b) and 0.567 (e). Candidate genes overlapped with significantly selected regions are marked by black color. (**c, d, f**) Manhattan plots of selective analysis by XP-CLR, and the thresholds are XP-CLR ≥ 8.986 (c), 9.723 (d) and 11.723 (f). Candidate genes overlapped with regions which were significantly selected by XPCLR & ln(π ratio)/ln(2), XPCLR & ln(π ratio)/ln(2) & *iHS*, XPCLR & ln(π ratio)/ln(2) & HKA and XPCLR & ln(π ratio)/ln(2) & *iHS* & HKA are marked by grey, orange, blue and red colors, respectively. (**g**) The patterns of genotypes of *IQGAP* region among one improved breed (EFR, high milk yield) and three low milk yielding breeds (SFK, FIN and DPS). Source data are provided as a Source Data file.

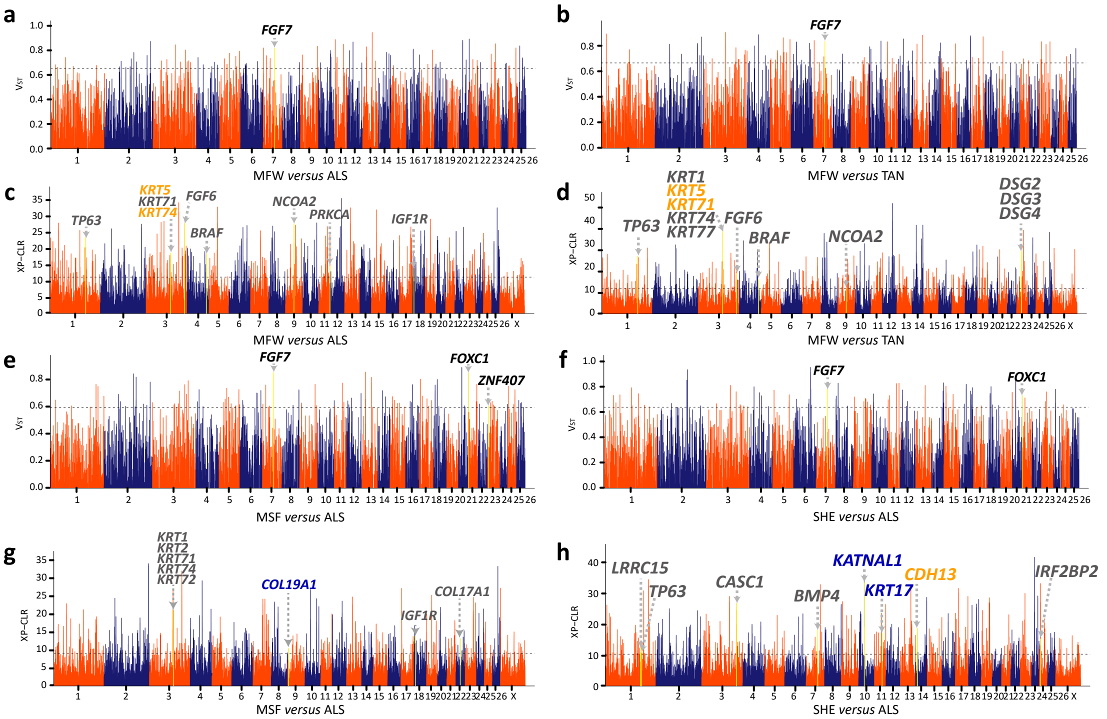

Supplementary Figure 15. Genome-wide screening and functional annotations of selected regions associated with wool related traits. whole-genome copy number variations (CNVs) and SNPs data were used to perform selective analysis through comparing fine, semi-fine and super-fine wool breeds (SHE, MSF and MFW) with two coarse wool breeds (ALS and TAN). (**a, b, e, f**) Statistic V_{ST} in four comparisons is plotted against the position of CNVs on each of the 26 autosomes, and the thresholds are V_{ST} value ≥ 0.649 (a), 0.666 (b), 0.596 (e) and 0.640 (f). Candidate genes overlappedwith significantly selected regions are marked by black color. (**c, d, g, h**) Manhattan plots of selective analysis by XP-CLR and the thresholds are XP-CLR ≥ 11.320 (c), 11.996 (d), 9.234 (g) and 10.401 (h). Candidate genes overlapped with regions which were significantly selected by XPCLR & ln(π ratio)/ln(2), XPCLR & ln(π ratio)/ln(2) & *iHS* and XPCLR & ln(π ratio)/ln(2) & HKA are marked by grey, orange and blue colors, respectively. Source data are provided as a Source Data file.

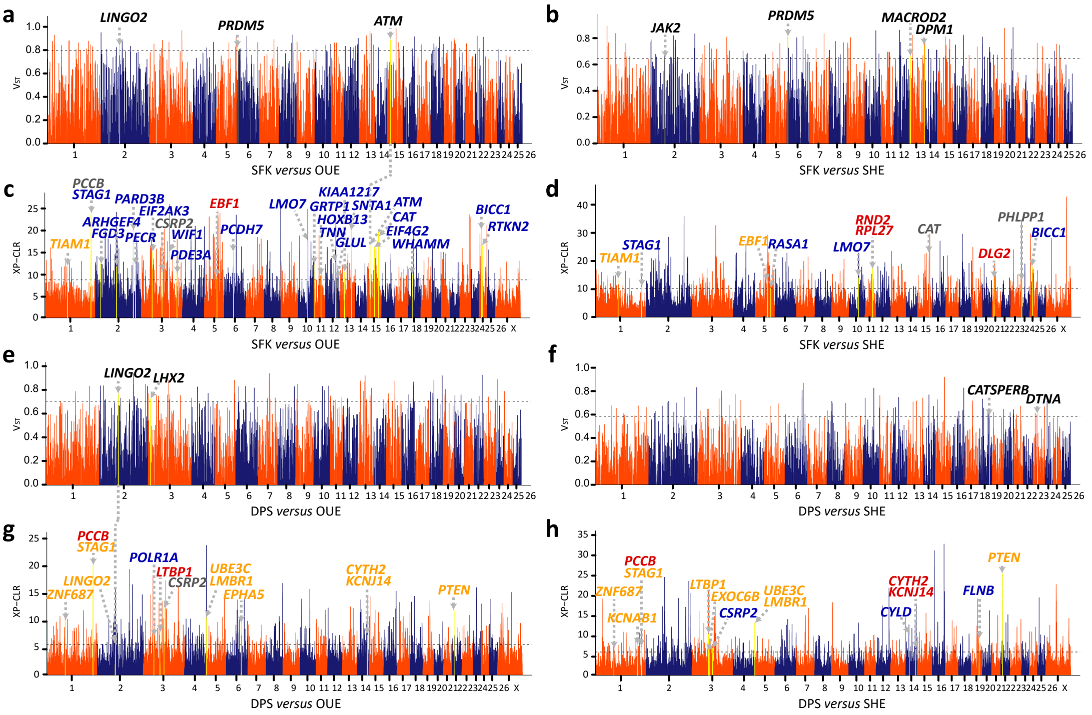

Supplementary Figure 16. Genome-wide screening and functional annotations of selected regions associated with the traits of meat production and growth rate. whole-genome copy number variations (CNVs) and SNPs data were used to perform selective analysis through pairwise comparisons between two improved breeds with large body size, fast growing and good quality of meat (SFK and DPS) and two landrace breeds with small body size (OUE and SHE). (**a, b, e, f**) Statistic V_{ST} in the four comparisons is plotted against the position of CNVs on each of the 26 autosomes, and the thresholds are V_{ST} value ≥0.797 (a), 0.644 (b), 0.704 (e) and 0.580 (f). Candidate genes overlapped with significantly selected regions are marked by black color. (**c, d, g, h**) Manhattan plots of selective analysis by XP-CLR and the thresholds are XP-CLR ≥ 8.807 (c), 10.406 (d), 5.723 (g) and 5.944 (h). Candidate genes overlapped with regions which were significantly selected by XPCLR & ln(π ratio)/ln(2), XPCLR & ln(π ratio)/ln(2) & *iHS*, XPCLR & ln(π ratio)/ln(2) & HKA and XPCLR & ln(π ratio)/ln(2) & *iHS* & HKA are marked by grey, orange, blue and red colors, respectively. Source data are provided as a Source Data file.

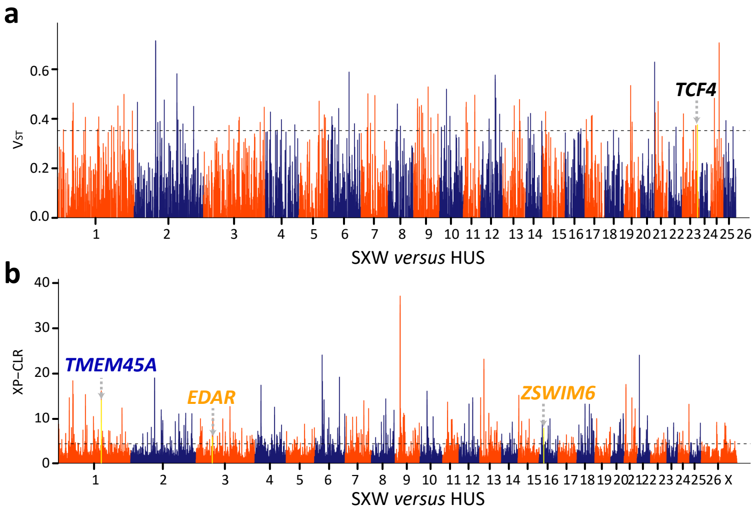

Supplementary Figure 17. Genome-wide screening and functional annotations of selected regions associated with horn related trait. whole-genome copy number variations (CNVs) and SNPs data were used to perform selective analysis through comparing horned sheep (SXW) with polled sheep (HUS). (**a**) Statistic V_{ST} in the comparison is plotted against the position of CNVs on each of the 26 autosomes, and the thresholds is V_{ST} value ≥ 0.351. Candidate genes overlapped with significantly selected regions are marked by black color. (**b**) Manhattan plots of selective analysis by XP-CLR and the thresholds is XP-CLR ≥ 4.488. Candidate genes overlapped with regions which were significantly selected by XPCLR & ln(π ratio)/ln(2) & *iHS* and XPCLR & ln(π ratio)/ln(2) & HKA are marked by orange and blue colors, respectively. Source data are provided as a Source Data file.

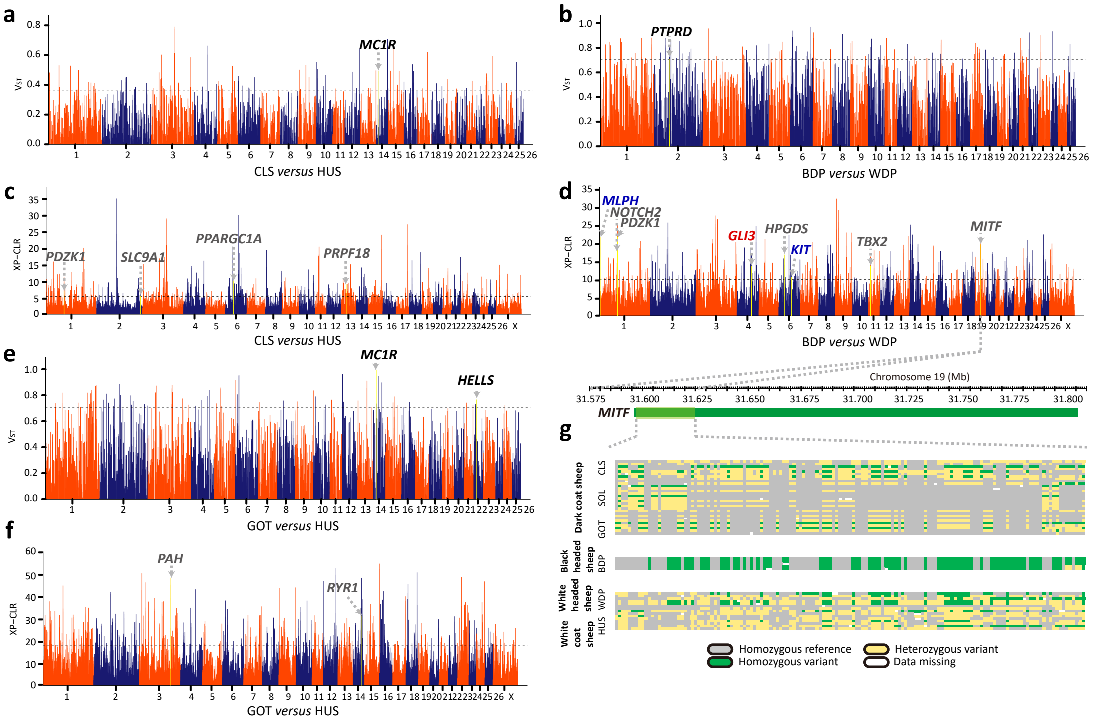

Supplementary Figure 18. Genome-wide screening and functional annotations of selected regions associated with coat colors. Through comparing black headed Dorper sheep (BDP) with white headed Dorper sheep (WDP) and dark coat sheep (CLS, GOT) with white coat sheep (HUS), whole-genome copy number variations (CNVs) and SNPs data were used to perform selective analysis. (**a, b, e**) Statistic V_{ST} in the three comparisons is plotted against the position of CNVs on each of the 26 autosomes, and the thresholds are V_{ST} value ≥ 0.365 (a), 0.701 (b) and 0.705 (e). Candidate genes overlapped with significantly selected regions are marked by black color. (**c, d, f**) Manhattan plots of selective analysis by XP-CLR and the thresholds are XP-CLR ≥ 5.509 (c), 10.146 (d) and 18.187 (f). Candidate genes overlapped with regions which were significantly selected by XPCLR & ln(π ratio)/ln(2), XPCLR & ln(π ratio)/ln(2) & HKA and ln(π ratio)/ln(2) & *iHS* & HKA are marked by grey, blue and red colors, respectively. (**g**) The patterns of genotypes of *MITF* region among dark coat sheep (CLS, GOT and SOL), black headed Dorper sheep (BDP), white headed Dorper sheep (WDP) and white coat sheep (HUS). Source data are provided as a Source Data file.

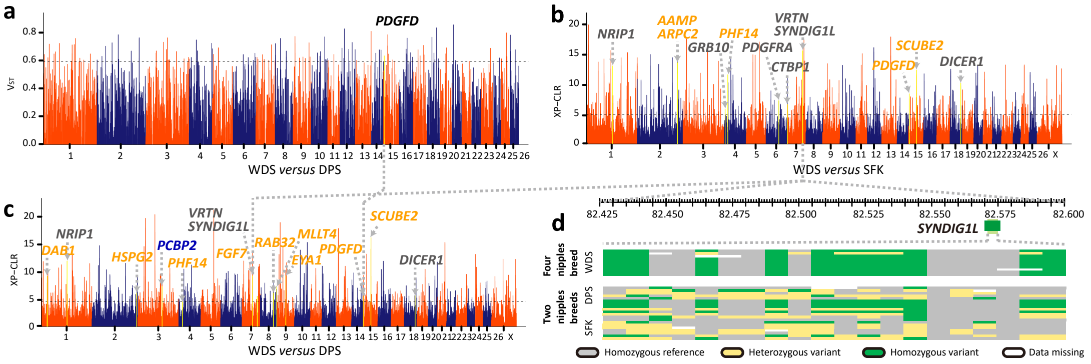

Supplementary Figure 19. Genome-wide screening and functional annotations of selected regions associated with number of nipples. Whole-genome copy number variations (CNVs) and SNPs data were used to perform selective analysis through pairwise comparisons between one landrace breed with four nipples (WDS) and two improved breeds with two nipples (SFK and DPS). (**a**) Statistic V_{ST} in the comparisons is plotted against the position of CNVs on each of the 26 autosomes, and the thresholds are V_{ST} value ≥ 0.594. Candidate genes overlapped with significantly selected regions are marked by black color. (**b, c**) Manhattan plots of selective analysis by XP-CLR and the thresholds are XP-CLR ≥ 5.005 (b) and 4.710 (c). Candidate genes overlapped with regions which were significantly selected by XPCLR & ln(π ratio)/ln(2), XPCLR & ln(π ratio)/ln(2) & *iHS* and XPCLR & ln(π ratio)/ln(2) & HKA are marked by grey, orange and blue colors, respectively. (**d**) The patterns of genotypes of *SYNDIG1L* region among one landrace breed with four nipples (WDS) and two improved breeds with two nipples (SFK and DPS). Source data are provided as a Source Data file.

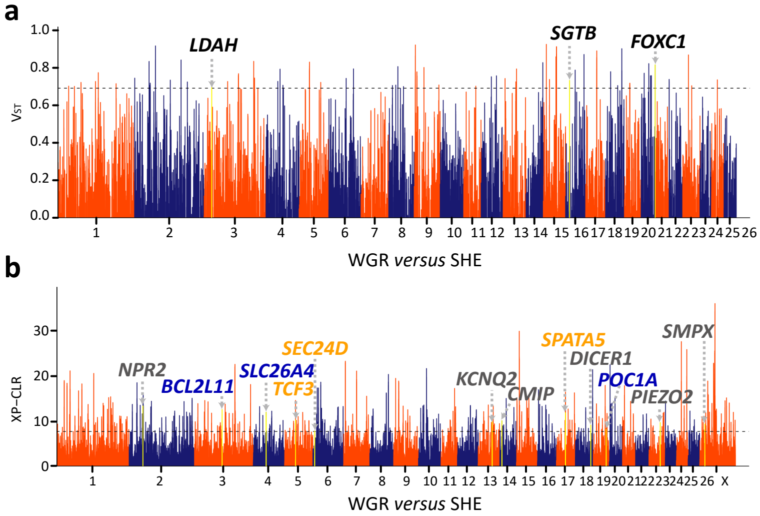

Supplementary Figure 20. Genome-wide screening and functional annotations of selected regions associated with ear size. Through comparing large ear-sized sheep (WGR) with small ear-sized sheep (SHE), whole-genome copy number variations (CNVs) and SNPs data were used to perform selective analysis. (**a**) Statistic V_{ST} in the comparison is plotted against the position of CNVs on each of the 26 autosomes, and the thresholds is V_{ST} value ≥ 0.691. Candidate genes overlapped with significantly selected regions are marked by black color. (**b**) Manhattan plots of selective analysis by XP-CLR and the threshold is XP-CLR ≥ 7.776. Candidate genes overlapped with regions which were significantly selected by XPCLR & ln(π ratio)/ln(2), XPCLR & ln(π ratio)/ln(2) & *iHS* and XPCLR & ln(π ratio)/ln(2) & HKA are marked by grey, orange and blue colors, respectively. Source data are provided as a Source Data file.

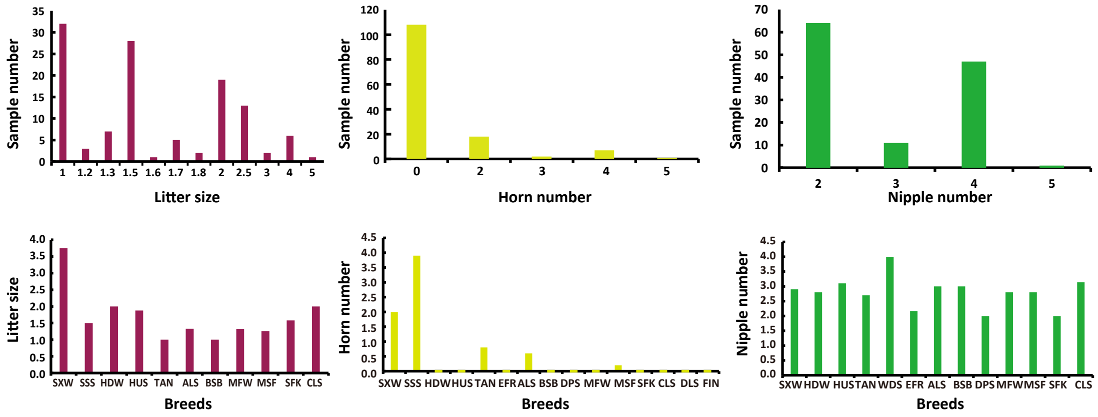

Supplementary Figure 21. Histograms showing the distribution of sample number in different phenotypic values for three traits and the average phenotypic values in different breeds. Breeds analyzed for litter size include SXW, SSS, HDW, HUS, TAN, ALS, BSB, MFW, MSF, SFK and CLS; for number of horns include SXW, SSS, HDW, HUS, TAN, EFR, ALS, BSB, DPS, MFW, MSF, SFK, CLS, DLS and FIN; for number of nipples include SXW, HDW, HUS, TAN, WDS, EFR, ALS, BSB, DPS, MFW, MSF, SFK and CLS. The sample size for each breed in the bar is ten. Source data are provided as a Source Data file.

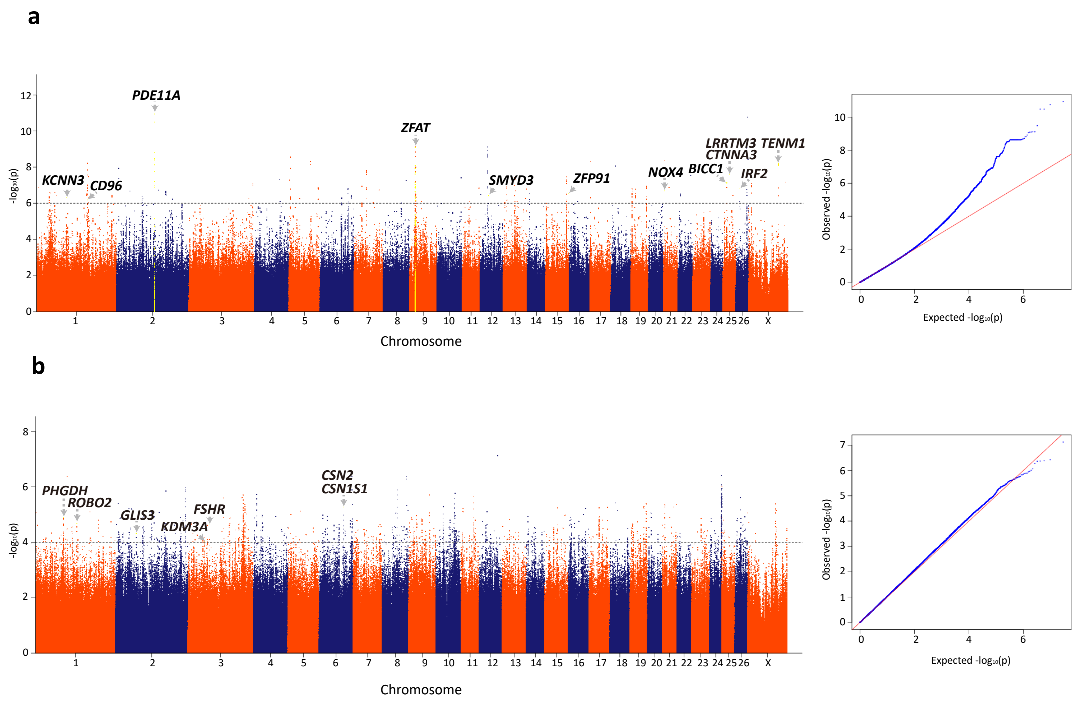

Supplementary Figure 22. GWAS analyses of sheep litter size and number of nipples traits based on the compressed MLM approach using SNP data. (**a**) Manhattan plot and quantile-quantile plot for litter size. The horizontal dashed line indicates the genome-wide significance threshold ($-\log_{10}P = 6$). (**b**) Manhattan plot and quantile-quantile plot for number of nipples. The horizontal dashed line indicates the genome-wide significance threshold ($-\log_{10}P = 4$).

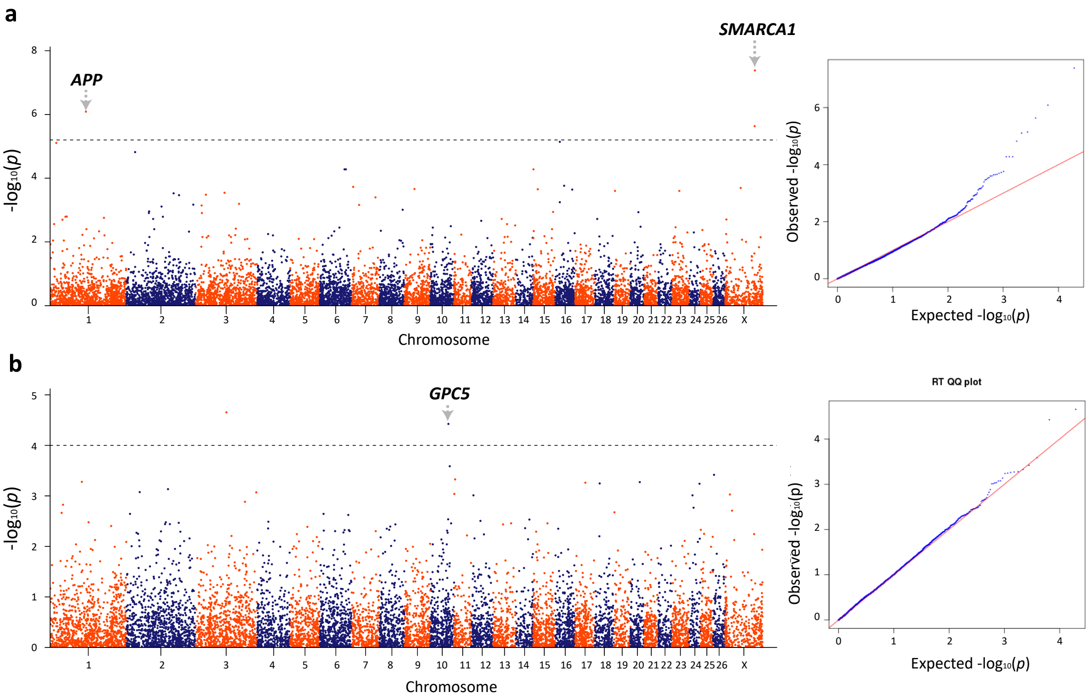

Supplementary Figure 23. GWAS analysis of sheep litter size and number of nipples trait based on copy number variation (CNV). (**a**) Manhattan plot and quantile-quantile plot for litter size. The horizontal dashed line indicates the genome-wide significance threshold ($-\log_{10}(0.05/\text{total CNVs}) = 5.286$). (**b**) Manhattan plot and quantile-quantile plot for number of nipples. The horizontal dashed line indicates the genome-wide significance threshold ($-\log_{10}P = 4$).

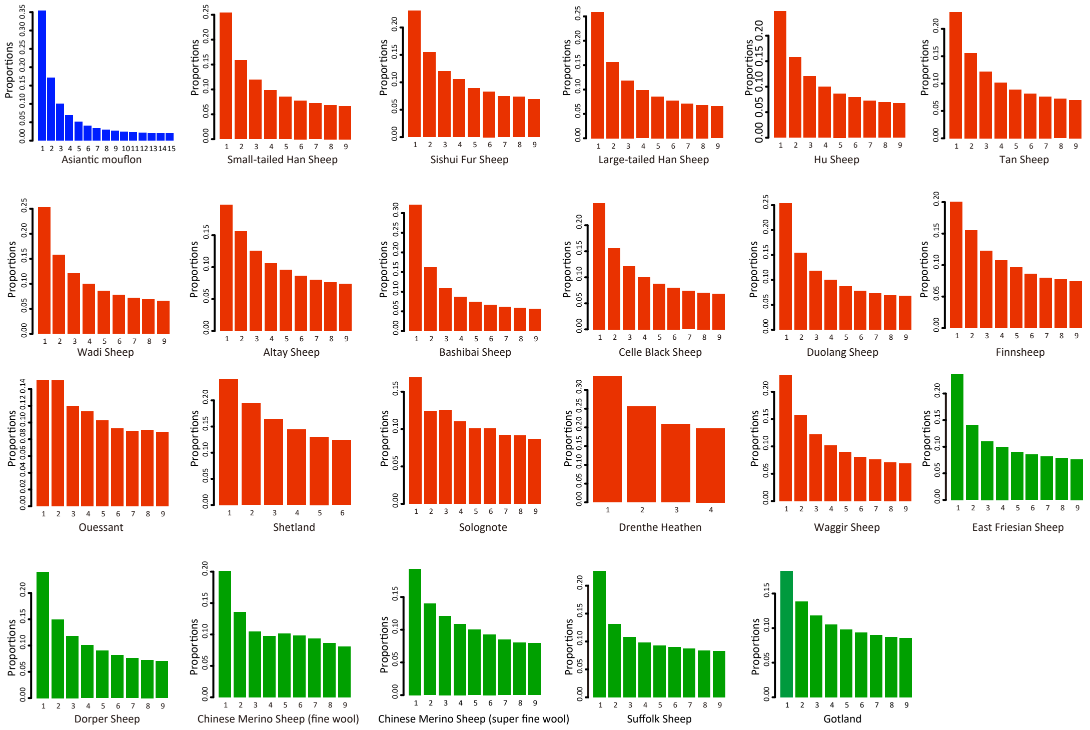

Supplementary Figure 24. Shape of the site frequency spectrum (SFS) among Asiatic mouflon and 22 populations of domestic sheep. The number for each population is shown in Supplementary Data 1. Source data are provided as a Source Data file.

Supplementary Tables

Supplementary Table 1. Validation of SNP calling by Sanger sequencing.

Gene	Chromosome	SNP position	Forward primer	Reverse primer	Identified individuals	Total sequencing individuals	Validated rate
KCNN3	1	103,418,790	TTCAGTTACTTTCCCCTTAT	CTCCGAGACAGAACCACA	14	14	100.00%
IQGAP3	1	104,967,138	GGGCTCCCTCTAGTTTCAG	CTCCTTCCACAGCACCAA	19	19	100.00%
IQGAP3	1	104,978,153	GACAGAAGAGGGAAACCAG	TGCAGTATTTAAGAGCGAAC	18	21	85.71%
IQGAP3	1	104,982,318	GAGTTAGGTGGGAGGTGTTG	TGCAGTATTTAAGAGCGAAC	21	21	100.00%
NRIP1	1	140,419,488	AACAAAGGGAAGACTGAAA	TCTAATGGCTCGCTGTAA	20	21	95.24%
SPAG8	2	52,480,881	AGGCTGTTCTGTCCTGGGTT	GGGCGGCTTCAAGGTTCT	20	20	100.00%
GLIS3	2	71,714,831	GTGAACATAGTCAAGGTAG	GACAATAAGAAGCTGGTA	20	20	100.00%
GLIS3	2	71,716,173	CCGAAATTATCAACTGAG	ACCTTATGCTGGACCTTG	14	16	87.50%
GLIS3	2	71,720,662	GGAAAACTGACTGGGATA	TCTTGCTAACAGATGCTC	21	21	100.00%
ARHGEF4	2	113,520,927	AGAACGGGCTCCAATAAC	CCTGTGCTCAGTATGTCCC	21	22	95.45%
ARHGEF4	2	113,521,020	AGAACGGGCTCCAATAAC	CCTGTGCTCAGTATGTCCC	22	22	100.00%
ARHGEF4	2	113,521,140	AGAACGGGCTCCAATAAC	CCTGTGCTCAGTATGTCCC	22	22	100.00%
ARHGEF4	2	113,521,429	AGAACGGGCTCCAATAAC	CCTGTGCTCAGTATGTCCC	22	22	100.00%
ARHGEF4	2	113,521,474	AGAACGGGCTCCAATAAC	CCTGTGCTCAGTATGTCCC	22	22	100.00%
ARHGEF4	2	113,521,731	AGAACGGGCTCCAATAAC	CCTGTGCTCAGTATGTCCC	22	22	100.00%
ARHGEF4	2	113,521,763	AGAACGGGCTCCAATAAC	CCTGTGCTCAGTATGTCCC	21	22	95.45%
ARHGEF4	2	113,521,810	AGAACGGGCTCCAATAAC	CCTGTGCTCAGTATGTCCC	22	22	100.00%
ARHGEF4	2	113,522,473	AGGAACTGTGCTCCGACA	CCTCACTCCCAAGAAGGT	15	18	83.33%
ARHGEF4	2	113,522,488	AGGAACTGTGCTCCGACA	CCTCACTCCCAAGAAGGT	16	19	84.21%
ARHGEF4	2	113,522,731	AGGAACTGTGCTCCGACA	CCTCACTCCCAAGAAGGT	18	21	85.71%

ARHGEF4	2	113,522,788	AGGAACTGTGCTCCGACA	CCTCACTCCCAAGAAGGT	17	21	80.95%
ARHGEF4	2	113,523,015	AGGAACTGTGCTCCGACA	CCTCACTCCCAAGAAGGT	18	21	85.71%
ARHGEF4	2	113,523,051	AGGAACTGTGCTCCGACA	CCTCACTCCCAAGAAGGT	18	21	85.71%
ARHGEF4	2	113,523,193	AGGAACTGTGCTCCGACA	CCTCACTCCCAAGAAGGT	17	21	80.95%
PDE11A	2	131,322,022	GAACAGGATGCTTGAAAA	AATGAGGTGGTAGATGCC	21	21	100.00%
PDE11A	2	131,323,179	AAAACCAGCATGAGTTCC	ACCAAGCGTAATCCAAAC	20	22	90.91%
PDE11A	2	131,323,506	AAAACCAGCATGAGTTCC	ACCAAGCGTAATCCAAAC	20	22	90.91%
HOXD1	2	132,835,651	GACTTTGTTGCACCCATG	AGCCTAACCCTCCCCTAA	22	22	100.00%
LRP1B	2	168,384,233	AATCAAATCTTGGTCATG	CAGTTTCTAATCCAGTTC	5	5	100.00%
LRP1B	2	168,392,544	CATTCATTTCCTCTATTTTC	TATCTTTTGCTTCGGTTA	21	21	100.00%
KDM3A	3	58,018,079	ATGTAAACCCTTAGTTGC	CTATGTGCCAGACTATGTT	21	21	100.00%
KRT72	3	133,462,372	ATCATCTCCAGTCGGGTCAGC	TGTCAATGAAGGAGGCGAAC	22	22	100.00%
KRT72	3	133,472,160	AAGCATTTACCACCAATCAG	TTGCCCTCAACCTTCCTA	22	22	100.00%
KRT72	3	133,473,817	AAATAGACAATGGAGGGTG	CATGGCGAGATGGGTGAG	14	18	77.78%
KRT74	3	133,486,008	GAGGTTACAACTTCAGGCTTAG	CACCCTTTCCATCCACAAT	22	22	100.00%
MSRB3	3	154,039,306	GCTATGGGATTCGCTTTA	AACTCGGCATCCTTGTCT	21	21	100.00%
LEMD3	3	154,244,699	TGACTACCATCTGGCTCC	GGCAACACTAACTCAACG	20	21	95.24%
LEMD3	3	154,244,764	TGACTACCATCTGGCTCC	GGCAACACTAACTCAACG	20	20	100.00%
LEMD3	3	154,245,273	TGACTACCATCTGGCTCC	GGCAACACTAACTCAACG	22	22	100.00%
LEMD3	3	154,246,255	GTCTCCAATGTCCACCCA	TTTCGTTCCTTGCTCCTG	22	22	100.00%
GRM3	4	33,682,552	ACAGAAACCTTCCACCTT	GCAGATACTTCCCACATAC	22	22	100.00%
BMPR1B	6	29,292,593	CGATGGCTCTGGTGAATC	GAGAAGGCTGACTGTGGC	19	20	95.00%
BMPR1B	6	29,292,891	CGATGGCTCTGGTGAATC	GAGAAGGCTGACTGTGGC	17	20	85.00%
BMPR1B	6	29,462,931	GAGAAAAGTCACCTCCAG	CAGCACCTAACATACACC	22	22	100.00%
FAM184B	6	37,070,391	CTCCTGCTGATGCTGTTCC	GGTTCTTCTGGCTGCTTTC	22	22	100.00%
FAM184B	6	37,073,418	TGGACACTGGGTGATGGC	GGATAGGATAGTTGTAGGGTTT	13	13	100.00%

PDE6B	6	116,764,581	GTGGTGTCGTTCCAAAATCG	GGAGGGCTCCCCTGAGCT	22	22	100.00%
TSHR	7	89,369,706	GGGCAGTAACAGGAAATG	ACACGGTATAGTCGTAATGGTT	22	22	100.00%
ZFAT	9	20,144,813	AAAATATCAACAGCCCAAAA	TGTAGACACCTGCCCTCA	21	21	100.00%
NSMAF	9	37,497,720	AGGGTTTATAACTCGTCT	GTTGCTGTTACTATTTGAG	22	22	100.00%
BRCA2	10	28,882,034	GTGCTGTATGCAGGTGGT	GTAAAGAAGGGCTTGACT	18	22	81.82%
BRCA2	10	28,882,998	ACTTCACTATTGTCTTGCTT	AACTTTGAAATGACCACC	20	22	90.91%
BRCA2	10	28,883,288	ACTTCACTATTGTCTTGCTT	AACTTTGAAATGACCACC	22	22	100.00%
BRCA2	10	28,883,312	ACTTCACTATTGTCTTGCTT	AACTTTGAAATGACCACC	22	22	100.00%
BRCA2	10	28,884,883	AGAGTTTGGGTTGACAGT	TAGCTCCAAGGATCTTCT	22	22	100.00%
RXFP2	10	29,432,742	TATCTGAGTCCAGTATTCTT	ACATCTAATTCTAGCGTG	22	22	100.00%
RXFP2	10	29,432,846	TATCTGAGTCCAGTATTCTT	ACATCTAATTCTAGCGTG	22	22	100.00%
MACROD2	13	8,414,586	CTGCTGTCATCCCTCCTG	TGCCCTTCCTCTGTCAAT	21	22	95.45%
PDGFD	15	3,904,410	TTACAGTAGCCATAGAAAA	AAATCCTGGATTACATTG	22	22	100.00%
BCO2	15	21,953,019	CTGACAAGTCCCCATAGC	ATACCCAACAAGGCAAAA	21	22	95.45%
LOC101108252	15	47,790,483	TGTCTTTATCAACTCTGCCTAC	TTGTGGTGGAAACCCTTA	22	22	100.00%
LOC101108252	15	47,790,995	TGTCTTTATCAACTCTGCCTAC	TTGTGGTGGAAACCCTTA	22	22	100.00%
ZFP91	15	78,943,932	TGTTAGTGTATTTCGGGTGT	CTCTTGGTCAATCTTACTTCAT	21	22	95.45%
NOX4	21	5,586,762	TTTTGGAATGAGGGAGGT	CGCTCAGTGGCAAAGAAC	15	21	71.43%
PCDH15	22	4,789,466	GTCTATAACTTTCAAGGGGACT	CACTGAGGGCAGGGCTAC	21	22	95.45%
SETBP1	23	44,663,758	CTTTTGAGCTTTTGTGCA	GTGGAACCTGGATGGAAT	22	22	100.00%
TRAP1	24	3,026,268	TGAGCACGCCCCTCCTTC	CTGCAGACACGGTGGGTTA	20	20	100.00%
GPC3	X	95,635,238	GTTTGAAAGGGCACAAGA	AGGAAGGAAAGCCAGAGT	22	22	100.00%

Supplementary Table 2. Annotation of SNPs in the two domestic (native, improved) and one wild (Asiatic mouflon) sheep groups.

Category	Groups		
	Mouflons	Landraces	Improved breeds
Sample size	16	172	60
Total number of SNPs	23,269,423	14,382,975	14,008,509
Intergenic (%)	66.1%	66.4%	66.4%
Intronic (%)	32.1%	31.9%	31.9%
Exonic (%)	0.580%	0.548%	0.551%
Synonymous (%)	0.374%	0.356%	0.355%
Non-synonymous (%)	0.204%	0.190%	0.193%
Non-synonymous/Synonymous	0.55	0.53	0.54
Splicing (%)	0.00110%	0.00101%	0.00099%
Upstream (%)	0.569%	0.540%	0.538%
Downstream (%)	0.572%	0.549%	0.543%
Upstream to downstream (SNP number)	1796	983	965
Unknowns (%)	0.1%	0.0%	0.0%
Ts/Tv	2.613	2.624	2.622

Supplementary Table 3. Details on linkage disequilibrium (LD, mesured as r^2) and LD extent to half the maximum value of r^2 for the 23 sheep populations, the three sheep groups (wild, native and improved) and the four groups from different geographic regions.

Category	Populations/Groups	Min	Median	Mean	Max	LD extent (kb)
The 23 populations	MOU	0.0962	0.0974	0.1014	0.4812	2.8
	SXW	0.1459	0.1501	0.1646	0.6203	8.9
	SSS	0.1656	0.1705	0.1849	0.6345	10.5
	HDW	0.1437	0.1487	0.1631	0.6197	8.9
	HUS	0.1434	0.1498	0.1646	0.6237	9.3
	TAN	0.1653	0.1702	0.1848	0.6356	10.4
	WDS	0.1483	0.1531	0.1677	0.6228	9.3
	EFR	0.2021	0.2139	0.2273	0.6610	14.8
	ALS	0.1896	0.1926	0.2065	0.6481	11.9
	BSB	0.1505	0.1542	0.1664	0.5979	8.2
	DPS	0.1826	0.1881	0.2006	0.6316	10.9
	MFW	0.2425	0.2502	0.2626	0.6781	20.5
	MSF	0.2164	0.2240	0.2365	0.6591	15.5
	SFK	0.2373	0.2472	0.2599	0.6833	19.8
	CLS	0.1590	0.1645	0.1780	0.6278	9.8
	DLS	0.1574	0.1623	0.1760	0.6262	9.6
	FIN	0.1743	0.1826	0.1971	0.6472	11.6
	OUE	0.3075	0.3322	0.3468	0.7608	68.3
	SHE	0.2534	0.2623	0.2757	0.6959	23.9
	SOL	0.2773	0.2960	0.3100	0.7307	38.2
	GOT	0.2346	0.2535	0.2685	0.7063	25.2
	DRS	0.3218	0.3307	0.3425	0.7261	61.2
	WGR	0.1597	0.1638	0.1787	0.6352	10.1
The three groups	Wild group	0.0962	0.0974	0.1014	0.4812	2.8
	Native group	0.1828	0.1890	0.2031	0.6484	12.1
	Improved group	0.2216	0.2307	0.2436	0.6698	17.1
The four groups from different geographic regions	The Middle East group	0.1413	0.1448	0.1592	0.6293	8.2
	Asian group	0.1584	0.1627	0.1768	0.6268	9.8
	African group	0.1420	0.1474	0.1636	0.6431	9.1
	European group	0.2518	0.2635	0.2767	0.6969	25.1

Supplementary Table 4. Details on LD (measured as r^2) within each chromosome and LD extend to half the maximum value of r^2 for the three (native, improved and wild) sheep groups.

Category	Chromosome	Min	Median	Mean	Max	LD extent (kb)
Wild sheep (i.e., Asiatic mouflon)	Chr. 1	0.0956	0.0972	0.1017	0.4868	3.2
	Chr. 2	0.0937	0.0968	0.1010	0.4894	3.0
	Chr. 3	0.0960	0.0980	0.1022	0.4924	3.0
	Chr. 4	0.0955	0.0973	0.1013	0.4857	3.0
	Chr. 5	0.0952	0.0976	0.1012	0.4803	2.7
	Chr. 6	0.0946	0.0969	0.1011	0.4873	3.0
	Chr. 7	0.0953	0.0977	0.1012	0.4832	2.6
	Chr. 8	0.0960	0.0986	0.1021	0.4859	2.8
	Chr. 9	0.0933	0.0971	0.1012	0.4809	3.1
	Chr. 10	0.0911	0.0978	0.1027	0.4817	3.0
	Chr. 11	0.0954	0.0985	0.1023	0.4750	2.4
	Chr. 12	0.0950	0.0972	0.1008	0.4738	2.7
	Chr. 13	0.0953	0.0978	0.1014	0.4870	2.6
	Chr. 14	0.0970	0.0997	0.1031	0.4730	2.5
	Chr. 15	0.0947	0.0979	0.1017	0.4848	2.8
	Chr. 16	0.0943	0.0968	0.1005	0.4807	2.7
	Chr. 17	0.0926	0.0977	0.1011	0.4668	2.7
	Chr. 18	0.0943	0.0970	0.1006	0.4779	2.6
	Chr. 19	0.0948	0.0974	0.1006	0.4729	2.5
	Chr. 20	0.0924	0.0967	0.1003	0.4700	2.5
	Chr. 21	0.0962	0.0985	0.1017	0.4609	2.5
	Chr. 22	0.0939	0.0978	0.1033	0.4731	2.6
	Chr. 23	0.0967	0.0989	0.1027	0.4707	2.7
	Chr. 24	0.0937	0.0968	0.0995	0.4698	2.1
	Chr. 25	0.0885	0.0964	0.0995	0.4628	2.5
	Chr. 26	0.0910	0.0971	0.1004	0.4626	2.6
Native sheep group	Chr. 1	0.1852	0.1915	0.2068	0.6513	13.4
	Chr. 2	0.1871	0.1948	0.2105	0.6619	13.8
	Chr. 3	0.1884	0.1967	0.2120	0.6656	13.7
	Chr. 4	0.1849	0.1918	0.2068	0.6563	12.8
	Chr. 5	0.1827	0.1888	0.2024	0.6476	11.7
	Chr. 6	0.1865	0.1945	0.2089	0.6561	13.2
	Chr. 7	0.1818	0.1882	0.2020	0.6524	11.2
	Chr. 8	0.1879	0.1942	0.2086	0.6571	12.3
	Chr. 9	0.1873	0.1953	0.2093	0.6483	13.4
	Chr. 10	0.1938	0.2025	0.2174	0.6567	15.6
	Chr. 11	0.1834	0.1923	0.2053	0.6527	11.6
	Chr. 12	0.1810	0.1900	0.2031	0.6435	11.4
	Chr. 13	0.1906	0.1982	0.2124	0.6647	13.0
	Chr. 14	0.1843	0.1903	0.2030	0.6445	11.3
	Chr. 15	0.1915	0.1987	0.2139	0.6590	13.7

	Chr. 16	0.1835	0.1916	0.2053	0.6497	11.7
	Chr. 17	0.1860	0.1926	0.2050	0.6355	11.7
	Chr. 18	0.1820	0.1887	0.2024	0.6478	11.8
	Chr. 19	0.1850	0.1920	0.2049	0.6463	11.8
	Chr. 20	0.1841	0.1918	0.2033	0.6328	10.5
	Chr. 21	0.1899	0.1977	0.2104	0.6366	13.5
	Chr. 22	0.1896	0.1971	0.2102	0.6419	12.6
	Chr. 23	0.1840	0.1911	0.2038	0.6354	11.9
	Chr. 24	0.1846	0.1932	0.2044	0.6442	9.9
	Chr. 25	0.1783	0.1891	0.2011	0.6332	11.2
	Chr. 26	0.1825	0.1892	0.2015	0.6336	11.9
Improved sheep group	Chr. 1	0.2187	0.2279	0.2413	0.6670	17.4
	Chr. 2	0.2294	0.2371	0.2506	0.6825	19.3
	Chr. 3	0.2349	0.2422	0.2568	0.6919	19.9
	Chr. 4	0.2176	0.2302	0.2429	0.6737	17.5
	Chr. 5	0.2099	0.2216	0.2344	0.6644	14.8
	Chr. 6	0.2322	0.2362	0.2499	0.6766	17.9
	Chr. 7	0.2211	0.2257	0.2383	0.6694	15.2
	Chr. 8	0.2152	0.2229	0.2364	0.6698	15.5
	Chr. 9	0.2272	0.2322	0.2450	0.6634	17.9
	Chr. 10	0.2247	0.2361	0.2487	0.6715	21.1
	Chr. 11	0.2144	0.2242	0.2362	0.6690	14.8
	Chr. 12	0.2289	0.2324	0.2450	0.6685	16.6
	Chr. 13	0.2225	0.2359	0.2485	0.6836	17.8
	Chr. 14	0.2222	0.2353	0.2475	0.6723	16.6
	Chr. 15	0.2235	0.2263	0.2412	0.6699	17.1
	Chr. 16	0.2149	0.2311	0.2437	0.6686	15.6
	Chr. 17	0.2086	0.2188	0.2298	0.6454	14.7
	Chr. 18	0.2391	0.2428	0.2553	0.6787	19.1
	Chr. 19	0.2024	0.2145	0.2270	0.6583	13.7
	Chr. 20	0.2119	0.2228	0.2336	0.6497	13.5
	Chr. 21	0.2241	0.2238	0.2357	0.6476	16.4
	Chr. 22	0.2168	0.2213	0.2329	0.6524	14.6
	Chr. 23	0.2331	0.2432	0.2555	0.6663	19.4
	Chr. 24	0.2063	0.2202	0.2299	0.6537	11.7
	Chr. 25	0.2222	0.2317	0.2425	0.6565	16.2
	Chr. 26	0.2136	0.2275	0.2392	0.6510	16.0

Supplementary Table 5. Detailed information of permutation tests for QTL overlaps.

Methods	Category	Number of sweep/CNVs/signals	Number of overlapping QTLs	Number of overlapping sweep	Number of more than observed overlapping sweep	*P* value
XP-CLR, π ratio and *iHS*	Domestication	54	204(shorter than 100M)	49	0	0
	Reproduction	29	3(shorter than 100M)	5	38	0.0038
	Milk related trait	12	1(shorter than 100M)	1	287	0.0287
	Tail related trait	36	7(shorter than 100M)	6	128	0.0128
	Wool related trait	24	3(shorter than 100M)	2	2254	0.2254
	Disease resistance	13	7(62M longest)	5	4	0.0004
	Body size and meat and growth related trait	19	20(shorter than 100M)	11	0	0
XP-CLR, π ratio and HKA	Domestication	47	125(shorter than 100M)	30	2	0.0002
	Reproduction	6	1(74M longest)	2	4	0.0004
	Milk related trait	8	4(shorter than 100M)	3	0	0
	Tail related trait	16	2(shorter than 100M)	2	128	0.0128
	Disease resistance	23	12(76M longest)	13	0	0
	Body size and meat and growth related trait	42	32(shorter than 100M)	23	0	0
V_{ST} based on CNVs	Domestication	137	238(shorter than 100M)	120	0	0
	Reproduction	530	6(74M longest)	32	8706	0.8706
	Milk related trait	371	38(shorter than 100M)	77	5532	0.5532
	Horn related trait	387	1(33M longest)	6	2301	0.2301
	Tail related trait	516	11(shorter than 100M)	32	10000	1
	Wool related trait	485	13(shorter than 100M)	72	2073	0.2073
	Disease resistance	428	73(84M longest)	239	0	0
	Body size and meat and growth related trait	475	119(shorter than 100M)	320	0	0
	Coat color	486	1(6.6M longest)	4	74	0.0074

Global F_{ST}	-	205	215(shorter than 100M)	131	5684	0.5684
GWAS based on SNPs	Litter size	600	5(74M longest)	121	0	0
	Horn number	988	1(33M longest)	87	0	0
	Nipple number	1969	4(14M longest)	31	5852	0.5852
GWAS based on CNVs	Horn number	6	1(33M longest)	1	26	0.0026

Supplementary Table 6. Thirty-six candidate genes associated with domestication in this study overlapping with two recent sheep studies (1, 2).

Symbol	Chromosome	Start	End	Methods
IGF2BP2	1	199,458,969	199,620,851	XPCLR, π ratio, HKA
RFX3	2	71,030,087	71,358,098	XPCLR, π ratio
SLC11A1	2	219,375,536	219,386,591	XPCLR, π ratio
CTDSP1	2	219,391,840	219,397,956	XPCLR, π ratio
PPP6C	3	10,694,625	10,732,536	XPCLR, π ratio
SCAI	3	10,733,659	10,852,123	XPCLR, π ratio
MBOAT2	3	18,134,066	18,195,721	XPCLR, π ratio, *iHS*, HKA
KITLG	3	124,487,491	124,594,363	XPCLR, π ratio, HKA
AGMO	4	23,622,538	24,021,242	XPCLR, π ratio, *iHS*, HKA
TRIM24	4	101,912,553	102,027,287	XPCLR, π ratio, HKA
RASGRF2	5	78,324,210	78,490,622	XPCLR, π ratio, *iHS*
LEF1	6	17,147,295	17,268,176	XPCLR, π ratio, *iHS*
HERC5	6	36,127,176	36,174,237	XPCLR, π ratio, *iHS*, HKA
KDR	6	70,477,415	70,524,370	XPCLR, π ratio, HKA
CPLX1	6	116,495,489	116,536,815	XPCLR, π ratio, HKA
PDSS2	8	29,677,799	29,959,570	XPCLR, π ratio
FLT1	10	31,814,061	32,020,993	XPCLR, π ratio
FBXO39	11	24,710,948	24,720,142	XPCLR, π ratio
XAF1	11	24,721,002	24,733,827	XPCLR, π ratio
UPF2	13	15,587,287	15,682,170	XPCLR, π ratio
HAO1	13	49,912,393	49,973,527	XPCLR, π ratio, *iHS*
RNF24	13	50,411,482	50,510,426	XPCLR, π ratio
IL18	15	21,868,663	21,893,266	XPCLR, π ratio, HKA
TEX12	15	21,896,461	21,901,658	XPCLR, π ratio, HKA
BCO2	15	21,905,805	21,973,313	XPCLR, π ratio, HKA
LOC101112255	15	21,909,862	21,910,675	XPCLR, π ratio, HKA
LOC101108252	15	47,790,046	47,790,999	XPCLR, π ratio
TRIP13	16	70,862,442	70,877,093	XPCLR, π ratio, *iHS*
ADAMTSL3	18	23,035,198	23,398,500	XPCLR, π ratio, *iHS*, HKA
PTPN9	18	32,176,758	32,249,831	XPCLR, π ratio
NKX2-1	18	45,530,212	45,534,128	XPCLR, π ratio
THOC7	19	37,637,112	37,655,593	XPCLR, π ratio
PPIL1	20	10,808,111	10,831,351	XPCLR, π ratio, HKA
GMDS	20	49,931,917	50,271,320	XPCLR, π ratio
GTF2I	24	32,681,983	32,763,945	XPCLR, π ratio, HKA
CUX1	24	34,643,893	34,887,512	XPCLR, π ratio, *iHS*

1. Naval-Sanchez, M. *et al.* Sheep genome functional annotation reveals proximal regulatory elements contributed to the evolution of modern breeds. *Nat. Commun.* **9**, 859 (2018).
2. Alberto, F.J. *et al*. Convergent genomic signatures of domestication in sheep and goats. *Nat. Commun.* **9**, 813 (2018).

Supplementary Table 7. Details on allele frequencies of the non-synonymous mutations located within the candidate genes identified in the domestication selective analysis.

Category	Gene ID	Chr.	Gene start	Gene end	SNP site	Reference allele	Mutant allele	Amino acid change	Breed	Mutant allele frequency
Photoreceptor development	*PDE6B*	6	116,758,387	116,786,025	116,764,581	G	T	Leucine/Methionine	MOU	0.66
									ALS	0
									HUS	0.05
									DRS	0
									DJI	0
									KAR	0
Yellow-fat	*BCO2*	15	21,905,805	21,973,313	21,953,019	A	T	Isoleucine/Phenylalanine	MOU	0.84
									ALS	0.2
									HUS	0.15
									DRS	0
									DJI	0
									KAR	0
Olfactory signal transduction	*LOC101108252*	15	47,790,046	47,790,999	47,790,483	A	G	Isoleucine/Methionine	MOU	0.63
									ALS	0.05
									HUS	0.05
									DRS	0.1
									DJI	0
									KAR	0
		15	47,790,046	47,790,999	47,790,995	T	C	Methionine/Threonine	MOU	0.63
									ALS	0.05
									HUS	0.05
									DRS	0.1

									DJI	0
									KAR	0
susceptibility gene for diabetes	*ADAMTSL3*	18	23,035,198	23,398,500	23,035,729	C	G	Glycine/Arginine	MOU	0.78
									ALS	0.1
									HUS	0
									DRS	0
									DJI	0
									KAR	0
Regulating cell fate and organ morphogenesis; controlling puberty and reproductive capability of females	*NKX2-1*	18	45,530,212	45,534,128	45,533,821	C	T	Glycine/Serine	MOU	0.38
									ALS	0.05
									HUS	0.17
									DRS	0
									DJI	0
									KAR	0

Supplementary Table 8. Gene annotation of putative CNVs experiencing domestication sweeps with top 1% V_{ST} values on each chromosome.

Chr.	CNVs			Genes			
	Start	End	V_{ST}	Symbol	Start	End	Annotation
1	170,539,806	170,540,600	0.613699	*BBX*	170,447,198	170,735,877	HMG box transcription factor BBX isoform X1
	275,276,500	275,276,691	0.520044	*SGOL1*	275,263,565	275,280,686	LOW QUALITY PROTEIN: shugoshin-like 1 isoform X1
2	52,624,434	52,624,900	0.55711	*LOC101116452*	52,623,154	52,624,931	P antigen family member 4-like
	72,841,608	72,842,216	0.514613	*JAK2*	72,812,001	72,927,328	tyrosine-protein kinase JAK2 isoform X1
3	56,725,801	56,726,467	0.491521	*TCF7L1*	56,694,077	56,885,168	LOW QUALITY PROTEIN: transcription factor 7-like 1 isoform X1
	169,653,301	169,653,900	0.727052	*ANO4*	169,412,537	169,857,047	anoctamin-4 isoform X1
6	39,798,401	39,799,100	0.700875	*SLIT2*	39,616,737	40,019,700	slit homolog 2 protein isoform X1
	55,555,534	55,555,701	0.601007	*ARAP2*	55,384,477	55,565,899	arf-GAP with Rho-GAP domain, ANK repeat and PH
	55,555,701	55,556,465	0.691927				domain-containing protein 2 isoform X1
7	18,155,601	18,156,531	0.771065	*THSD4*	17,722,301	18,412,109	thrombospondin type-1 domain-containing protein 4 isoform X1
	57,751,201	57,751,490	0.510661	*FGF7*	57,740,594	57,804,130	fibroblast growth factor 7 isoform X1
				FAM227B	57,652,046	57,867,901	protein FAM227B isoform X1
8	87,235,000	87,235,169	0.50973	*PDE10A*	87,039,739	87,395,395	cAMP and cAMP-inhibited cGMP 3',5'-cyclic phosphodiesterase 10A isoform X1
	88,093,700	88,093,800	0.528698	*RPS6KA2*	87,946,415	88,109,559	ribosomal protein S6 kinase alpha-2 isoform X1
10	31,538,801	31,539,086	0.688916	*MTUS2*	31,211,953	31,595,495	microtubule-associated tumor suppressor candidate 2 isoform X2
	70,323,513	70,323,982	0.49451	*LOC101106781*	70,243,533	70,484,912	multidrug resistance-associated protein 4-like isoform X1
	70,465,010	70,465,500	0.584062				
11	62,088,606	62,089,300	0.621318	*HELZ*	61,943,713	62,094,438	probable helicase with zinc finger domain isoform X1
12	26,203,417	26,203,800	0.58428	*DNAH14*	26,103,140	26,512,095	dynein heavy chain 14, axonemal isoform X1
13	9,710,901	9,711,101	0.521106	*KIF16B*	9,536,194	9,848,679	LOW QUALITY PROTEIN: kinesin-like protein KIF16B isoform X1
	9,711,101	9,711,201	0.541588				
	9,711,201	9,712,400	0.628514				

	9,712,400	9,712,600	0.560777				
	9,712,600	9,712,700	0.633812				
	9,712,700	9,712,800	0.544496				
	15,322,301	15,323,000	0.52556	*USP6NL*	15,213,628	15,403,904	LOW QUALITY PROTEIN: USP6 N-terminal-like protein isoform X1
	24,011,403	24,011,887	0.642079	*KIAA1217*	23,929,045	24,433,187	sickle tail protein homolog isoform X1
14	33,576,726	33,581,900	0.57501	*CMTM2*	33,570,766	33,591,977	CKLF-like MARVEL transmembrane domain-containing protein 2
	39,150,221	39,150,662	0.558073	*HYDIN*	39,025,231	39,372,668	hydrocephalus-inducing protein homolog isoform X1
	58,144,801	58,162,400	0.634225	*LOC101111827*	58,149,458	58,154,548	cationic amino acid transporter 3-like
15	1,676,135	1,676,400	0.563409	*GRIA4*	1,439,067	2,098,596	glutamate receptor 4 isoform X2
	21,910,101	21,910,500	0.584207	*LOC101112255*	21,909,862	21,910,675	prefoldin subunit 4
				BCO2	21,905,805	21,973,313	beta,beta-carotene 9',10'-oxygenase isoform X1
	29,032,938	29,033,300	0.511624	*CCDC84*	29,030,736	29,038,549	coiled-coil domain-containing protein 84 isoform X1
20	8,225,501	8,225,867	0.502329	*GRM4*	8,215,696	8,322,629	metabotropic glutamate receptor 4 isoform X1
24	10,812,744	10,812,994	0.571334	*SNX29*	10,673,482	11,267,171	sorting nexin-29 isoform X1
	41,454,620	41,455,953	0.726184	*LOC105604882*	41,449,927	41,456,334	LOW QUALITY PROTEIN: cytochrome P450 2W1 isoform X1

Supplementary Table 9. The frequency of SVs overlapped with the area of 57 most plausible domesticated genes in Asiatic mouflon and domestic sheep (Dutch Drenthe Heathen, East-Asian Hu, Central-Asian Altay, African Djiallonke and Middle Eastern Karakul sheep).

Gene ID	Spcies	Frequency	Chr.	SV start	SV end	Length (bp)	Type
RFX3	Asiatic mouflon	0.63	2	71,134,116	71,134,842	727	DEL
	Domestic sheep	0.19					
AGMO	Asiatic mouflon	0.31	4	23,647,481	23,647,914	434	DEL
	Domestic sheep	0.05					
CAMK4	Asiatic mouflon	0.19	5	107,670,987	65,615,952	42,055,034	TRA
	Domestic sheep	0.05					
BCO2	Asiatic mouflon	0.81	15	21,909,861	21,910,683	823	DEL
	Domestic sheep	0.10					
LOC101112255	Asiatic mouflon	0.81	15	21,909,861	21,910,683	823	DEL
	Domestic sheep	0.10					
ADAMTSL3	Asiatic mouflon	1.00	18	23,071,408	23,072,887	1480	DEL
	Domestic sheep	0.24					
GTF2I	Asiatic mouflon	0.31	24	32,681,983	22,658,775	10,023,207	TRA
	Domestic sheep	0.00					
SGCZ	Asiatic mouflon	0.06	26	21,352,113	2,037,278	19,314,834	TRA
	Domestic sheep	0.41					
SGCZ	Asiatic mouflon	0.56	26	21,448,862	21,449,346	485	DEL
	Domestic sheep	0.14					
SGCZ	Asiatic mouflon	0.81	26	21,524,745	21,525,062	318	DEL
	Domestic sheep	0.33					

Supplementary Table 10. Overlaps between putatively selected domestication CNVs and the candidate genes associated with domestication identified by XP-CLR, π ratio and *iHS* or HKA.

Gene ID	Chr.	Gene start	Gene end	CNV start	CNV end	Length (bp)	Species	Sample ID	Type
USP6NL	13	15,213,628	15,403,904	15,322,301	15,323,000	700	Asiatic mouflon	266	Deletion
								267	Deletion
								273	Deletion
								SH.19	Deletion
								TH.1	Deletion
								YZ.11	Deletion
								YZ.9	Deletion
BCO2	15	21,905,805	21,973,313	21,910,101	21,910,500	400	Altay Sheep	AL109	Duplication
							Asiatic mouflon	266	Deletion
								271	Deletion
								273	Deletion
								TH.1	Deletion
								TH.2	Deletion
								KR.6	Deletion
								SH.7	Deletion
								YZ.12	Deletion
								SH.19	Deletion
								SH.20	Deletion
LOC101112255	15	21,909,862	21,910,675	21,910,101	21,910,500	400	Altay Sheep	AL109	Duplication
							Asiatic mouflon	266	Deletion
								271	Deletion
								273	Deletion
								TH.1	Deletion

TH.2	Deletion
KR.6	Deletion
SH.7	Deletion
YZ.12	Deletion
SH.19	Deletion
SH.20	Deletion

Supplementary Table 11. Overlapped genes identified by the global F_{ST} and the domestication-associated and trait-associated selective sweep analyses.

Chr.	Region start	Region end	F_{ST}	Genes	Gene start	Gene end	Category in the selective analysis	Functions
5	78,400,001	78,475,000	0.286966	*RASGRF2*	78,324,210	78,490,622	Domestication	Response to endoplasmic reticulum stress
10	31,925,001	32,000,000	0.2749725	*FLT1*	31,814,061	32,020,993	Domestication	Angiogenesis and vasculogenesis
11	24,700,001	24,750,000	0.278838	*FBXO39*	24,710,948	24,720,142	Domestication	SCF-dependent proteasomal ubiquitin-dependent protein catabolic process
11	24,700,001	24,750,000	0.278838	*XAF1*	24,721,002	24,733,827	Domestication	Apoptosis signaling pathway
20	49,950,001	50,125,000	0.2950622	*GMDS*	49,931,917	50,271,320	Domestication	Cell proliferation
4	68,700,001	69,050,000	0.2946663	*HOXA11*	68,817,583	68,824,465	Domestication	Fat tail formation
6	116,750,001	116,825,000	0.291294	*PDE6B*	116,758,387	116,786,025	Domestication	Photoreceptor development
13	56,275,001	56,400,000	0.3585945	*EDN3*	56,295,795	56,320,163	Domestication	Pigmentation
13	62,725,001	62,850,000	0.3058535	*RALY*	62,780,163	62,789,951	Domestication	Coat color
7	89,275,001	89,500,000	0.3624278	*TSHR*	89,198,176	89,372,207	Reproduction	Puberty
17	68,775,001	68,825,000	0.278827	*LIF*	68,790,962	68,797,781	Reproduction	Fertility
20	17,250,001	17,400,000	0.2796714	*VEGFA*	17,291,023	17,305,051	Reproduction	Follicular development
24	33,900,001	33,975,000	0.282448	*HIP1*	33,774,906	33,908,567	Reproduction	Ovarian development
1	103,300,001	103,375,000	0.2783155	*ADAR*	103,284,562	103,335,914	Horn related trait; Reproduction	Osteoblast differentiation; Embryonic erythropoiesis
10	29,350,001	29,600,000	0.3231027	*RXFP2*	29,434,933	29,509,159	Horn related trait; Reproduction	Hornless; Follicle development
7	57,775,001	57,900,000	0.3461148	*FGF7*	57,740,594	57,804,130	Fat tail; Nipple number	White adipose development; Mammary gland development
7	89,275,001	89,500,000	0.3624278	*TSHR*	89,198,176	89,372,207	Fat tail; Ear size	Adipogenesis; Auditory development
15	3,825,001	3,900,000	0.295128	*PDGFD*	3,852,935	4,137,382	Fat tail; Nipple number	Fat tail (known); Breast cancer
19	11,725,001	11,800,000	0.277778	*XYLB*	11,725,661	11,766,807	Fat tail	Fat tail

19	11,725,001	11,800,000	0.277778	*ACVR2B*	11,794,562	11,802,479	Fat tail	Reduction in tail length
X	80,025,001	80,100,000	0.2878255	*MTM1*	80,063,927	80,154,168	Fat tail	Positive regulation of skeletal muscle tissue growth
1	195,575,001	195,650,000	0.280369	*TP63*	195,514,420	195,783,654	Wool related trait	Wool production
3	133,475,001	133,525,000	0.273539	*KRT74*	133,485,549	133,494,262	Wool related trait	Wool fineness
3	133,475,001	133,525,000	0.273539	*KRT71*	133,502,784	133,510,767	Wool related trait	Wool
10	30,600,001	30,725,000	0.3055643	*KATNAL1*	30,714,583	30,759,391	Wool related trait	Fiber diameter
25	7,225,001	7,500,000	0.366624	*IRF2BP2*	7,398,685	7,401,002	Wool related trait	Composition and structure of fleece
4	5,150,001	5,200,000	0.286234	*GRB10*	5,085,423	5,265,803	Nipple number	Mammary development
7	82,550,001	82,625,000	0.3002715	*SYNDIG1L*	82,570,893	82,576,311	Nipple number	Teat number
2	52,450,001	52,500,000	0.277103	*NPR2*	52,481,117	52,499,438	Ear size	Auditory circuit assembly
3	153,975,001	154,125,000	0.3255094	*MSRB3*	154,036,231	154,218,498	Ear size	Ear size
3	154,200,001	154,250,000	0.283056	*LEMD3*	154,247,456	154,342,991	Ear size	Ear size
3	154,200,001	154,250,000	0.283056	*MSRB3*	154,036,231	154,218,498	Ear size	Ear size
13	53,075,001	53,550,000	0.3421976	*KCNQ2*	53,417,912	53,461,353	Ear size	Inner ear development

Supplementary Table 12. Detailed information on pairwise comparisons for detecting selective signals associated with particular traits.

Traits	Category	Populations	Comparisons
Reproduction	Prolificacy	SXW, FIN, HUS, GOT, WDS	SXW *versus* TAN; FIN *versus* TAN; HUS *versus* TAN; GOT *versus* TAN; WDS *versus* TAN
	Low litter size	TAN	
Milk production	High yielding	EFR	EFR *versus* FIN; EFR *versus* SFK; EFR *versus* DPS
	Low yielding	FIN, SFK, DPS	
Tail Type	Fat-tailed	HDW, ALS, BSB	HDW *versus* TAN; HDW *versus* SHE; ALS *versus* SHE; BSB *versus* SHE; DRS *versus* SHE
	Long wooly tail	DRS	
	Thin-tailed	SHE, TAN	
Horn type	3~5 horns	SSS	SSS *versus* SXW; SSS *versus* TAN; SXW *versus* HUS; HUS *versus* SXW
	0~2 horns	SXW (2 horns), TAN (0~2 horns)	
	polled	HUS	
Wool fineness	Fine wool (including super fine wool)	MFW, MSF, SHE	MFW *versus* TAN; MSF *versus* TAN; MFW *versus* ALS; MSF *versus* ALS; SHE *versus* ALS
	Coarse wool	ALS, TAN	
Nipple number	Four nipples	WDS	WDS *versus* DPS; WDS *versus* SFK
	Two nipples	DPS, SFK	
Meat production, growth rate trait and body size	Large body size	SFK, DPS	SFK *versus* OUE; SFK *versus* SHE; DPS *versus* OUE; DPS *versus* SHE
	Small body size	OUE, SHE	
Ear size	Big ear	WGR, DLS	WGR *versus* SHE; DLS *versus* SHE
	Small erect ear	SHE	
Coat color	White (with black head)	BDP	BDP *versus* WDP; CLS *versus* HUS; GOT *versus* HUS; SOL *versus* HUS
	White	WDP, HUS	
	Black	CLS	
	Brown	SOL	
	Grey (heads and legs are black)	GOT	

Supplementary Table 13. Summary information of the mRNA sequencing reads.

Breed	Sample ID	Raw reads	Raw base (bp)	Clean reads	Clean base (bp)	Mapping rate
	MFW1	57,470,856	8,620,628,400	53,924,496	8,088,674,400	91.37%
Chinese Merino Sheep (Thin-tailed)	MFW2	40,144,348	6,021,652,200	37,365,654	5,604,848,100	89.74%
	MFW3	53,617,716	8,042,657,400	50,969,968	7,645,495,200	90.03%
	SXW1	51,682,098	7,752,314,700	34,340,090	5,151,013,500	85.89%
Small-tailed Han sheep (Fat-tailed)	SXW2	47,779,584	7,166,937,600	37,497,370	5,624,605,500	89.46%
	SXW3	50,026,916	7,504,037,400	41,678,582	6,251,787,300	91.53%
	ALS1	53,408,362	8,011,254,300	51,125,456	7,668,818,400	92.12%
Altay sheep (Fat-rumped)	ALS2	56,267,788	8,440,168,200	51,589,002	7,738,350,300	92.70%
	ALS3	41,271,770	6,190,765,500	39,157,120	5,873,568,000	92.86%
	HDW1	41,124,686	6,168,702,900	38,465,972	5,769,895,800	93.00%
Large-tailed Han sheep (Fat-tailed)	HDW2	48,381,220	7,257,183,000	44,394,906	6,659,235,900	92.38%
	HDW3	43,624,888	6,543,733,200	38,708,920	5,806,338,000	94.79%

Supplementary Table 14. Genome-wide association signals using CNV data for litter size, horn number and nipple number.

Traits	Chromosome	CNV start	CNV end	$-\log_{10}(P\text{-value})$
Litter size	1	128,659,801	128,662,221	6.08522044
	X	106,688,516	106,690,665	7.383515874
	X	106,103,963	106,106,372	5.632224526
Horn number	8	39,329,453	39,333,124	7.307340846
	10	29,433,062	29,434,913	11.78637176
	18	11,930,893	11,932,685	6.776237023
	X	7,019,037	7,024,316	9.724792796
	X	7,032,677	7,033,661	9.724792796
	X	7,046,303	7,047,369	9.88710523
Nipple number	3	112,368,044	112,369,475	4.654685487
	10	64,952,791	64,955,595	4.424521298

Supplementary Table 15. Overlaps between GWAS signals based on CNV data and previously reported QTLs for the horn trait (1).

		GWAS		QTLs		
Chr.	CNV start	CNV end	$-\log_{10}$(*P*-value)	QTL Start	QTL End	Trait
10	29,433,062	29,434,913	11.78637	12,606,797	45,964,265	Horns QTL #12909

1. Hu, Z.L., Park, C.A. & Reecy, J.M. Building a livestock genetic and genomic information knowledgebase through integrative developments of Animal QTLdb and CorrDB. *Nucleic Acids Res.* **47,** D701–D710 (2019).

Supplementary Table 16. Overlaps between GWAS signals based on CNV data and putative selected CNVs using V_{ST} for litter size and horn number.

GWAS					Selective analysis		
Traits	Chromosome	CNV start	CNV end	$-\log_{10}(P\text{-value})$	Traits	CNV start	CNV end
Litter size	2	127,083,901	127,084,290	5.578198471	Reproduction	127,083,901	127,084,290
	16	31,318,104	31,319,372	5.249094838		31,318,104	31,319,372
Horn number	1	138,139,201	138,139,301	8.513343203	Horn related traits	138,139,201	138,139,301
	1	138,139,201	138,139,301	8.513343203		138,139,201	138,139,301
	9	28,900,801	28,901,001	11.71036214		28,900,801	28,901,001
	9	28,900,801	28,901,001	11.71036214		28,900,801	28,901,001
	23	47,748,401	47,749,200	7.146771508		47,748,401	47,749,200

Supplementary Table 17. Summary information of individuals used to construct neighbor-joining (NJ) tree based on the genetic groups.

Genetic groups	Populations	Individuals
African sheep (AFS)	BOG	BQ2602
	AFS	FKD2746
	MBS	Sc12
	YAN	Sn5
	WAD	Wad-Sn16
	UDA	uda-Sc3
	DJI	180b
	MOS	99
	SAH	163
	CAM	KA-07
East Asian sheep (EAS)	SXW	SXW30
	SSS	SSS45
	HDW	HDW51
	HUS	HU69
	TAN	TAN79
	TAN	TAN87
	ALS	AL119
	BSB	BSB123
	CLS	CLS234
	MAR	WDS102
Middle Eastern sheep (MES)	AWA	AWA-21
	HAS	HAM18
	MAZ	MAZ10
	GSS	Iran102
	GHE	Iran152
	AFH	Iran202
	SHA	Iran229
	MAK	MAK333
	MOH	MOG326
	KAR	Iran4265
European sheep (EUS)	FIN	FINN300
	FIN	FINN303
	OUE	Ouessant24802
	OUE	Ouessant24947
	SHE	Shetland23343
	SHE	Shetland23347
	SOL	Solognote24727
	SOL	Solognote24728
	DHS	DR1
	DHS	DR2
Asiatic Mouflon Sheep (AMS)	AMUF	266

AMUF	267
AMUF	272
AMUF	273
AMUF	KR.4
AMUF	KR.6
AMUF	YZ.9
AMUF	YZ.11
AMUF	SH.19
AMUF	SH.20

Supplementary Table 18. Reynolds genetic distances among four genetic groups of domestic sheep and the outgroup Asiatic mouflon.

**Genetic groups**	**Reynolds genetic distances**				
	AFS	EAS	MES	EUS	AMS
African sheep (AFS)	0.00000	0.06296	0.04963	0.08015	0.13713
East Asian sheep (EAS)	0.06296	0.00000	0.01062	0.05072	0.13135
Middle Eastern sheep (MES)	0.04963	0.01062	0.00000	0.04635	0.11803
European sheep (EUS)	0.08015	0.05072	0.04635	0.00000	0.13232
Asiatic Mouflon Sheep (AMS)	0.13713	0.13135	0.11803	0.13232	0.00000

Supplementary References

1. Wang, W. *et al*. Genomic variation in 3,010 diverse accessions of Asian cultivated rice. *Nature* **557,** 43–49 (2018).
2. Szpiech, Z.A. & Hernandez, R.D. Selscan: an efficient multithreaded program to perform EHH-based scans for positive selection. *Mol. Biol. Evol.* **31,** 2824–2827 (2014).
3. Jiang, Y. *et al*. The sheep genome illuminates biology of the rumen and lipid metabolism. *Science* **344,** 1168–1173 (2014).
4. Delaneau, O., Howie, B., Cox, A.J., Zagury, J.F. & Marchini, J. Haplotype estimation using sequencing reads. *Am. J. Hum. Genet.* **93,** 687–696 (2013).
5. Kijas, J.W. *et al*. Genome-wide analysis of the world's sheep breeds reveals high levels of historic mixture and strong recent selection. *PLoS Biol.* **10,** e1001258 (2012).
6. Hudson, R.R., Kreitman, M. & Aguadé, M. A test of neutral molecular evolution based on nucleotide data. *Genetics* **116,** 153–159 (1987).
7. Pagani, L. *et al*. Genomic analyses inform on migration events during the peopling of Eurasia. *Nature* **538,** 238–242 (2016).
8. Yan, G. *et al*. Genome sequencing and comparison of two nonhuman primate animal models, the cynomolgus and Chinese rhesus macaques. *Nat. Biotechnol.* **29,** 1019–1023 (2011).
9. Lalitha, S. Primer Premier 5. *Biotech Software & Internet Report* **1,** 6 (2000).
10. Hall, T.A. BioEdit: a user-friendly biological sequence alignment editor and

analysis program for Windows 95/98/NT. *Nucleic Acids Symposium Series* **41,** 95–98 (1999).

11. Schmittgen, T.D. & Livak, K.J. Analyzing real-time PCR data by the comparative C_T method. *Nat. Protoc.* **3,** 1101–1108 (2008).
12. D'haene, B., Vandesompele, J. & Hellemans, J. Accurate and objective copy number profiling using real-time quantitative PCR. *Methods* **50,** 262–270 (2010).
13. Miller, J.M., Moore, S.S., Stothard, P., Liao, X. & Coltman, D.W. Harnessing corss-species alignment to discover SNPs and generate a draft genome sequence of a bighorn sheep (*Ovis canadensis*). *BMC Genomics* **16,** 397 (2015).
14. Yang, J. *et al*. Whole-genome sequencing of native sheep provides insights into rapid adaptations to extreme environments. *Mol. Biol. Evol.* **33,** 2576–2592 (2016).
15. Stern, J.A., White, S.N. & Meurs, K.M. Extent of linkage disequilibrium in large-breed dogs: chromosomal and breed variation. *Mamm. Genome* **24,** 409–415 (2013).
16. Mdladla, K., Dzomba, E.F., Huson, H.J. & Muchadeyi, F.C. Population genomic structure and linkage disequilibrium analysis of South African goat breeds using genome-wide SNP data. *Anim. Genet.* **47,** 471–482 (2016).
17. Lan, D. *et al*. Genetic diversity, molecular phylogeny and selection evidence of Jinchuan yak revealed by whole-genome resequencing. *G3: Genes, Genomes, Genetics* **8,** 945–952 (2018).

18. Gray, M.M. *et al*. Linkage disequilibrium and demographic history of wild and domestic canids. *Genetics* **181,** 1493–1505 (2009).
19. Naval-Sanchez, M. *et al*. Sheep genome functional annotation reveals proximal regulatory elements contributed to the evolution of modern breeds. *Nat. Commun.* **9,** 859 (2018).
20. Muigai, A.W.T. & Hanotte, O. The origin of African sheep: archaeological and genetic perspectives. *Afr. Archaeol. Rev.* **30,** 39–50 (2013).
21. Zhao, Y.X. *et al*. Genomic reconstruction of the history of native Sheep reveals the peopling patterns of nomads and the expansion of early pastoralism in East Asia. *Mol. Biol. Evol.* **34,** 2380–2395 (2017).
22. Rocha, J., Chen, S. & Beja-Pereira, A. Molecular evidence for fat-tailed sheep domestication. *Trop. Anim. Health. Prod.* **43,** 1237–1243 (2011).
23. Lv, F.-H. *et al*. Mitogenomic meta-analysis identifies two phases of migration in the history of eastern Eurasian sheep. *Mol. Biol. Evol.* **32,** 2515–2533 (2015).
24. Scherf, B.D. World Watch List for Domestic Animal Diversity, 3rd edn (Food and Agriculture Organization of the United Nations, Rome, 2000).
25. Fariello, M.I. *et al*. Selection signatures in worldwide sheep populations. *PLoS One* **9,** e103813 (2014).
26. Chen, L. *et al*. Identification of sheep ovary genes potentially associated with off-season reproduction. *J. Genet. Genomics* **39,** 181–190 (2012).
27. Jafarpour, F. *et al*. Comparative dynamics of 5-methylcytosine reprogramming and *TET* family expression during preimplantation mammalian development in

mouse and sheep. *Theriogenology* **89,** 86–96 (2017).

28. Xu, S.S. *et al*. Genome-wide association analyses highlight the potential for different genetic mechanisms for litter size among sheep breeds. *Front. Genet.* **9,** 118 (2018).

29. Johnston, S.E., Bérénos, C., Slate, J. & Pemberton, J.M. Conserved genetic architecture underlying individual recombination rate variation in a wild population of soay sheep (*Ovis aries*). *Genetics* **203,** 583–598 (2016).

30. Yan, Y.B. *et al*. A pilot trial on the molecular pathophysiology of traumatic temporomandibular joint bony ankylosis in a sheep model. Part I: Expression of Wnt signaling. *J. Craniomaxillofac. Surg.* **42,** e15–e22 (2014).

31. Lantier, I. *et al*. Quantitative trait loci for resistance to infection in sheep using a live Salmonella Abortusovis vaccine. *Anim. Genet.* **43,** 632–635 (2012).

32. Seals, R.C., Urban, R.J., Sekar, N. & Veldhuis, J.D. Up-regulation of basal transcriptional activity of the cytochrome P450 cholesterol side-chain cleavage (*CYP11A*) gene by isoform-specific calcium-calmodulin-dependent protein kinase in primary cultures of ovarian granulosa cells. *Endocrinology* **145,** 5616–5622 (2004).

33. Yuan, Z. *et al*. Selection signature analysis reveals genes associated with tail type in Chinese indigenous sheep. *Anim. Genet.* **48,** 55–66 (2017).

34. Xu, S.S. *et al*. Genome-wide association analysis identifies the genetic basis of fat deposition in the tails of sheep (*Ovis aries*). *Anim. Genet.* **48,** 560–569 (2017).

35. Niu, Y. *et al*. Biallelic β-carotene oxygenase 2 knockout results in yellow fat in sheep via CRISPR/Cas9. *Anim. Genet.* **48,** 242–244 (2017).
36. Wood, S.H. *et al*. Binary switching of calendar cells in the pituitary defines the phase of the circannual cycle in mammals. *Curr. Biol.* **25,** 2651–2662 (2015).
37. Le, T.H., Christensen, O.F., Nielsen, B. & Sahana, G. Genome-wide association study for conformation traits in three Danish pig breeds. *Genet. Sel. Evol.* **49,** 12 (2017).
38. Gautier, M. *et al*. A whole genome Bayesian scan for adaptive genetic divergence in West African cattle. *BMC Genomics* **10,** 550 (2009).
39. Cheng, Z. *et al*. Acute bovine viral diarrhea virus infection inhibits expression of interferon tau-stimulated genes in bovine endometrium. *Biol. Reprod.* **96,** 1142–1153 (2017).
40. Reissmann, M. & Ludwig, A. Pleiotropic effects of coat colour-associated mutations in humans, mice and other mammals. *Semin. Cell Dev. Biol.* **24,** 576–586 (2013).
41. Wang, X. *et al*. Whole-genome sequencing of eight goat populations for the detection of selection signatures underlying production and adaptive traits. *Sci. Rep.* **6,** 38932 (2016).
42. Nazari-Ghadikolaei, A. *et al*. Genome-Wide Association studies identify candidate genes for coat color and mohair traits in the Iranian Markhoz goat. *Front. Genet.* **9,** 105 (2018).
43. Wilkinson, S. *et al*. Signatures of diversifying selection in European pig breeds.

PLoS Genet. **9,** e1003453 (2013).

44. Dreger, D.L., Parker, H.G., Ostrander, E.A. & Schmutz, S.M. Identification of a mutation that is associated with the saddle tan and black-and-tan phenotypes in Basset Hounds and Pembroke Welsh Corgis. *J. Hered.* **104,** 399–406 (2013).
45. Wang, M.S. *et al*. Positive selection rather than relaxation of functional constraint drives the evolution of vision during chicken domestication. *Cell Res.* **26,** 556–573 (2016).
46. vonHoldt, B.M. *et al*. Structural variants in genes associated with human Williams-Beuren syndrome underlie stereotypical hypersociability in domestic dogs. *Sci. Adv.* **3,** e1700398 (2017).
47. Zielak, A.E. *et al*. Identification of novel genes associated with dominant follicle development in cattle. *Reprod. Fertil. Dev.* **19,** 967–975 (2007).
48. Campbell, E.M., Nonneman, D.J., Kuehn, L.A. & Rohrer, G.A. Genetic variation in the mannosidase 2B2 gene and its association with ovulation rate in pigs. *Anim. Genet.* **39,** 515–519 (2008).
49. Okumu, L.A. *et al*. Temporal regulation of fibroblast growth factors and their receptors in the endometrium and conceptus during the pre-implantation period of pregnancy in cattle. *Reproduction* **147,** 825–834 (2014).
50. Redon, R. *et al*. Global variation in copy number in the human genome. *Nature* **444,** 444–454 (2006).
51. Berisha, B. *et al*. Expression of fibroblast growth factor 1 (*FGF1*) and *FGF7* in mature follicles during the periovulatory period after GnRH in the cow. *J. Reprod.*

Dev. **52,** 307–313 (2006).

52. Dickinson, R.E. *et al*. Involvement of the SLIT/ROBO pathway in follicle development in the fetal ovary. *Reproduction* **139,** 395–407 (2010).

53. Szewczuk, M. Association of a genetic marker at the bovine Janus kinase 2 locus (*JAK2/RsaI*) with milk production traits of four cattle breeds. *J. Dairy Res.* **82,** 287–292 (2015).

54. Wang, Z. *et al*. Genome-wide association study for wool production traits in a Chinese Merino sheep population. *PLoS One* **9,** e107101 (2014).

55. Cristancho, A.G. *et al*. Repressor transcription factor 7-like 1 promotes adipogenic competency in precursor cells. *Proc. Natl. Acad. Sci. USA* **108,** 16271–16276 (2011).

56. Hankir, M.K. *et al*. A novel thermoregulatory role for *PDE10A* in mouse and human adipocytes. *EMBO Mol. Med.* **8,** 796–812 (2016).

57. Ilardo, M.A. *et al*. Physiological and genetic adaptations to diving in Sea Nomads. *Cell* **173,** 569–580 (2018).

58. Wei, C. *et al*. Genome-wide analysis reveals population structure and selection in Chinese indigenous sheep breeds. *BMC Genomics* **16,** 194 (2015).

59. Fan, Q.C. *et al*. Identification of 19 loci for reproductive traits in a local Chinese chicken by genome-wide study. *Genet. Mol. Res.* **16,** gmr16019431 (2017).

60. Sevane, N., Dunner, S., Boado, A. & Cañon, J. Polymorphisms in ten candidate genes are associated with conformational and locomotive traits in Spanish Purebred horses. *J. Appl. Genet.* **58,** 355–361 (2017).

61. Mulsant, P. *et al*. Mutation in bone morphogenetic protein receptor-IB is associated with increased ovulation rate in Booroola Mérino ewes. *Proc. Natl. Acad. Sci. USA* **98,** 5104–5109 (2001).

62. Chen, Z., Yao, Y., Ma, P., Wang, Q. & Pan, Y. Haplotype-based genome-wide association study identifies loci and candidate genes for milk yield in Holsteins. *PLoS One* **13,** e0192695 (2018).

63. Wiedemar, N. & Drögemüller, C.A. 1.8-kb insertion in the 3′-UTR of *RXFP2* is associated with polledness in sheep. *Anim. Genet.* **46,** 457–461 (2015).

64. Kijas, J.W., Hadfield, T., Naval Sanchez, M. & Cockett, N. Genome-wide association reveals the locus responsible for four-horned ruminant. *Anim. Genet.* **47,** 258–262 (2016).

65. Liu, S.M. & Masters, D.G. Amino Acids Utilization for Wool Production (CAB International, Wallingford, 2003).

66. Ma, Q. *et al*. Genome-wide detection of copy number variation in Chinese indigenous sheep using an ovine high-density 600 K SNP array. *Sci. Rep*. **7,** 912 (2017).

67. Celichowski, P. *et al*. "Positive Regulation of RNA Metabolic Process" ontology group highly regulated in porcine oocytes matured in vitro: a microarray approach. *Biomed. Res. Int.* **2018,** 2863068 (2018).

68. Zhang, C. *et al*. A lung cancer gene *GPC5* could also be crucial in breast cancer. *Mol. Genet. Metab.* **103,** 104–105 (2011).

69. Hu, Z.L., Park, C.A. & Reecy, J.M. Building a livestock genetic and genomic

information knowledgebase through integrative developments of Animal QTLdb and CorrDB. *Nucleic Acids Res.* **47,** D701–D710 (2019).
