# Supplementary material for: Whole-genome resequencing of wild and domestic sheep identifies genes associated with morphological and agronomic traits
Source: Nat Commun. 2020 Jun 4;11:2815. doi: 10.1038/s41467-020-16485-1 (PMC7272655; doi:10.1038/s41467-020-16485-1)
Supplement: Supplementary file 3 — Description of Additional Supplementary Information [file 41467_2020_16485_MOESM3_ESM.pdf]

Description of Additional Supplementary Files

File Name: Supplementary Data 1-49
Description: The summary information of samples, sequencing and genetic variants, and results of selective tests, RNA-seq and genome-wide association analyses.
