# Supplementary material for: Whole-genome resequencing of wild and domestic sheep identifies genes associated with morphological and agronomic traits
Source: Nat Commun. 2020 Jun 4;11:2815. doi: 10.1038/s41467-020-16485-1 (PMC7272655; doi:10.1038/s41467-020-16485-1)
Supplement: Supplementary file 5 — Reporting summary [file 41467_2020_16485_MOESM5_ESM.pdf]

nature research

Corresponding author(s): Meng-Hua Li, Xin-Hua Wang

Last updated by author(s): Apr 18, 2020

# Reporting Summary

Nature Research wishes to improve the reproducibility of the work that we publish. This form provides structure for consistency and transparency in reporting. For further information on Nature Research policies, see Authors & Referees and the Editorial Policy Checklist .

## Statistics

For all statistical analyses, confirm that the following items are present in the figure legend, table legend, main text, or Methods section.

| n/a | Confirmed | |
|---|---|---|
| ☐ | ☒ | The exact sample size ($n$) for each experimental group/condition, given as a discrete number and unit of measurement |
| ☐ | ☒ | A statement on whether measurements were taken from distinct samples or whether the same sample was measured repeatedly |
| ☐ | ☒ | The statistical test(s) used AND whether they are one- or two-sided<br>*Only common tests should be described solely by name; describe more complex techniques in the Methods section.* |
| ☒ | ☐ | A description of all covariates tested |
| ☒ | ☐ | A description of any assumptions or corrections, such as tests of normality and adjustment for multiple comparisons |
| ☐ | ☒ | A full description of the statistical parameters including central tendency (e.g. means) or other basic estimates (e.g. regression coefficient) AND variation (e.g. standard deviation) or associated estimates of uncertainty (e.g. confidence intervals) |
| ☐ | ☒ | For null hypothesis testing, the test statistic (e.g. $F$, $t$, $r$) with confidence intervals, effect sizes, degrees of freedom and $P$ value noted<br>*Give P values as exact values whenever suitable.* |
| ☒ | ☐ | For Bayesian analysis, information on the choice of priors and Markov chain Monte Carlo settings |
| ☒ | ☐ | For hierarchical and complex designs, identification of the appropriate level for tests and full reporting of outcomes |
| ☒ | ☐ | Estimates of effect sizes (e.g. Cohen's $d$, Pearson's $r$), indicating how they were calculated |

*Our web collection on statistics for biologists contains articles on many of the points above.*

## Software and code

Policy information about availability of computer code

| | |
|---|---|
| Data collection | Burrows-Wheeler Aligner v.0.7.8, SAMtools v.1.3.1, Genome Analysis Toolkit (GATK) v.3.7, ANGSD v.0.915, ANNOVAR v.2013-06-21, CNVnator v.0.3.2, DELLY v.0.7.9, Manta v.1.6.0, SURVIVOR v.1.0.6, TreeBeST v.1.9.2, FigTree v.1.4.3, GCTA software v.1.24.2, ADMIXTURE v.1.23, PLINK v.1.07, Arlequin v3.5.2.2, PHYLIP v.3.695, vcftools v.0.1.14, pairwise sequentially Markovian coalescent (PSMC), SNeP v.1.0, XP-CLR v.1.0, Selscan v.1.2.0, GEMMA v.0.96, TASSEL v.5.0, BEDTools v2.26.0, HISAT v2.1.0, StringTie v2.0 and Ballgown package in R version 3.5.3 |
| Data analysis | This study used publicly available software which is referenced accordingly in the manuscript. No customised software was used. |

For manuscripts utilizing custom algorithms or software that are central to the research but not yet described in published literature, software must be made available to editors/reviewers. We strongly encourage code deposition in a community repository (e.g. GitHub). See the Nature Research guidelines for submitting code & software for further information.

## Data

Policy information about availability of data

All manuscripts must include a data availability statement. This statement should provide the following information, where applicable:
- Accession codes, unique identifiers, or web links for publicly available datasets
- A list of figures that have associated raw data
- A description of any restrictions on data availability

Raw sequencing data that support the findings of this study have been deposited to the NCBI BioProject database under accession PRJNA624020 [https://www.ncbi.nlm.nih.gov/bioproject/PRJNA624020]. The source data underlying Figs. 1, 3, 4, 5 and Supplementary Figs. 1-7, 9, 11–21 and 24 are provided as a Source Data file.

# Field-specific reporting

Please select the one below that is the best fit for your research. If you are not sure, read the appropriate sections before making your selection.

☒ Life sciences ☐ Behavioural & social sciences ☐ Ecological, evolutionary & environmental sciences

For a reference copy of the document with all sections, see nature.com/documents/nr-reporting-summary-flat.pdf

# Life sciences study design

All studies must disclose on these points even when the disclosure is negative.

| | |
|---|---|
| Sample size | No sample-size calculation was performed, but the sample size chosen was comparable to the previous literatures similarly reporting genomic resources for domesticated species. For instance, Naval-Sanchez et al. 2018 used 67 domestic sheep and 17 wild sheep for exploring genetic variation and genetic mechanism of domestication and selection. Alberto et al. 2018 used 13 wild Asiatic mouflon, 18 Bezoar ibex, 40 sheep and 44 goats to investigate Convergent genomic signatures of domestication in sheep and goats. Therefore, we think in our study, the samples size of 232 resequencing domestic sheep from all over the world and 16 resequencing wild sheep (Asiatic mouflon) to identify genes associated with morphological and agronomic traits is sufficient. |
| Data exclusions | No data were excluded from the analyses |
| Replication | All experiments (i.e., PCR, RT-PCR, qPCR and western blot) were repeated three times with similar results. |
| Randomization | 10,000 simulated data sets of genomic locations of selective sweeps/GWAS hits were randomly generated. The random sampling method was used to validate SNPs and CNVs. |
| Blinding | Blinding was not used because all analyses in the main text were performed on the premise that the samples are known. |

# Reporting for specific materials, systems and methods

We require information from authors about some types of materials, experimental systems and methods used in many studies. Here, indicate whether each material, system or method listed is relevant to your study. If you are not sure if a list item applies to your research, read the appropriate section before selecting a response.

## Materials & experimental systems

| n/a | Involved in the study | |
|---|---|---|
| ☐ | ☒ | Antibodies |
| ☒ | ☐ | Eukaryotic cell lines |
| ☒ | ☐ | Palaeontology |
| ☐ | ☒ | Animals and other organisms |
| ☒ | ☐ | Human research participants |
| ☒ | ☐ | Clinical data |

## Methods

| n/a | Involved in the study | |
|---|---|---|
| ☒ | ☐ | ChIP-seq |
| ☒ | ☐ | Flow cytometry |
| ☒ | ☐ | MRI-based neuroimaging |

## Antibodies

| | |
|---|---|
| Antibodies used | Rabbit Anti-beta Actin antibody (ab8227, Abcam), Anti-SCDGFB/PDGF-D antibody (ab181845, Abcam) and Goat Anti-Rabbit IgG H&L (ab205718, Abcam). |
| Validation | The antibodies ab8227, ab181854 and ab205718 have been successfully validated and applied for sheep as described in the main text and Figure 4. |

## Animals and other organisms

Policy information about studies involving animals; ARRIVE guidelines recommended for reporting animal research

| | |
|---|---|
| Laboratory animals | For DNA collection, all laboratory animals (248 samples) are adults. In 232 domestic sheep (Ovis aries), ten Small-tailed Han Sheep, ten Large-tailed Han Sheep, ten Hu Sheep, ten Tan Sheep, ten Wadi Sheep, ten Altay Sheep, ten Bashibai Sheep, ten Dorper Sheep, twenty Chinese Merino Sheep, ten Suffolk Sheep, ten Finnsheep, six Ouessant, four Shetland, seven Solognote, five Gotland, seven Waggir Sheep, seven Cele Black Sheep, five Duolang Sheep, six East Friesian Dairy Sheep, one Ghezel Sheep are female. Ten Sishui Fur Sheep, five Duolang Sheep, one Ouessant, three Shetland, three Solognote, five Gotland, five Drente Heathen, three Waggir Sheep, one Mbororo Sheep, one Grey-Shiraz Sheep, one Afshari Sheep, one Shal Sheep and one Karakul Sheep are male. The sexes of four East Friesian Dairy Sheep, three Cele Black Sheep, three Ouessant, one Bonga Sheep, one Afar Sheep, one Yankasa Sheep, one West African Dwarf Sheep, one Uda Sheep, one Djallonke Sheep, one Mossi Sheep, one Sahelian Sheep, one Cameroon Sheep, one Awassi Sheep, one Hamdani Sheep, one Mazekh Sheep, one Makui Sheep and one Moghani |

| | |
|---|---|
| | Sheep are not determined. In 16 wild sheep (Ovis orientalis), seven Asiatic muflon are female and nine Asiatic mouflon are male. For RNA collection, twelve samples are adults. Three Chinese Merino Sheep, three Altay sheep and three Large-tailed Han sheep are male. Three Small-tailed Han sheep are female. |
| Wild animals | In 16 wild sheep (Ovis orientalis), they are all adults. Seven Asiatic muflon are female and nine Asiatic mouflon are male. Blood from sixteen captive or recently hunted wild sheep (Asiatic mouflon) were used for the DNA extraction. After sampling, they were released at the sampling sites. |
| Field-collected samples | The study did not involve field collected samples. |
| Ethics oversight | All animal work was conducted according to a permit (No. IOZ13015) approved by the Committee for Animal Experiments of the Institute of Zoology, Chinese Academy of Sciences (CAS), China. For domestic sheep, animal sampling was also approved by local authorities where the samples were taken. For Asiatic mouflon, peripheral blood samples from 16 captive Asiatic mouflon were collected after receiving authorization for research from the Department of Environmental Protection in Iran (No. 93/34089). For two of the Asiatic mouflon samples from Shahr-e Kord, Iran, sampling procedure was also approved by the governorate of Chaharmahal and Bakhtiari of Iran (No. 97.32.43.33165). |

Note that full information on the approval of the study protocol must also be provided in the manuscript.
